# Co-Assembly of Cancer Drugs with Cyclo-HH Peptides: Insights from Simulations and Experiments

**DOI:** 10.1021/acsabm.3c01304

**Published:** 2024-03-13

**Authors:** Anastasia Vlachou, Vijay Bhooshan Kumar, Om Shanker Tiwari, Sigal Rencus-Lazar, Yu Chen, Busra Ozguney, Ehud Gazit, Phanourios Tamamis

**Affiliations:** † Artie McFerrin Department of Chemical Engineering, Texas A&M University, College Station, Texas 77843-3122, United States; ‡ The Shmunis School of Biomedicine and Cancer Research, George S. Wise Faculty of Life Sciences, Tel Aviv University, Tel Aviv 6997801, Israel; § Department of Materials Science and Engineering, Iby and Aladar Fleischman Faculty of Engineering, Tel Aviv University, Tel Aviv 6997801, Israel; ∥ Sagol School of Neuroscience, Tel Aviv University, Tel Aviv 6997801, Israel; ⊥ Department of Materials Science and Engineering, Texas A&M University, College Station, Texas 77843-3003, United States

**Keywords:** peptide self-assembly, peptide co-assembly with drugs, molecular dynamics simulations, cancer drugs, drug encapsulation

## Abstract

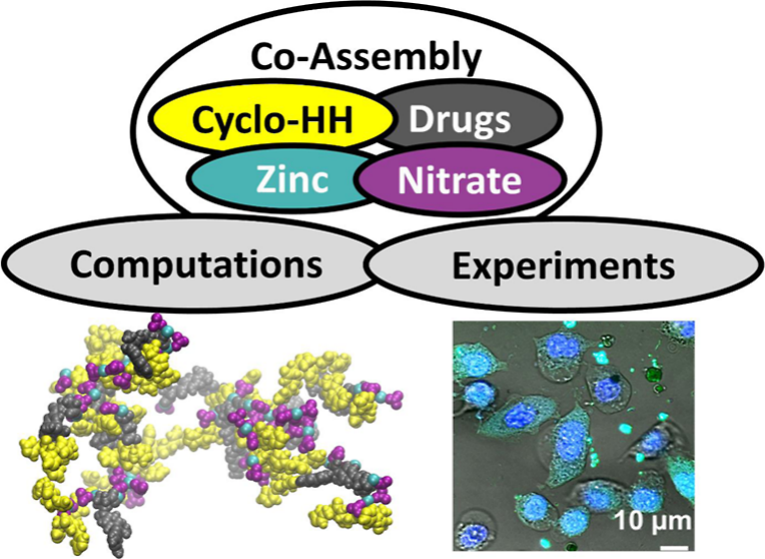

Peptide-based nanomaterials can serve as promising drug delivery agents, facilitating the release of active pharmaceutical ingredients while reducing the risk of adverse reactions. We previously demonstrated that Cyclo-Histidine-Histidine (Cyclo-HH), co-assembled with cancer drug Epirubicin, zinc, and nitrate ions, can constitute an attractive drug delivery system, combining drug self-encapsulation, enhanced fluorescence, and the ability to transport the drug into cells. Here, we investigated both computationally and experimentally whether Cyclo-HH could co-assemble, in the presence of zinc and nitrate ions, with other cancer drugs with different physicochemical properties. Our studies indicated that Methotrexate, in addition to Epirubicin and its epimer Doxorubicin, and to a lesser extent Mitomycin-C and 5-Fluorouracil, have the capacity to co-assemble with Cyclo-HH, zinc, and nitrate ions, while a significantly lower propensity was observed for Cisplatin. Epirubicin, Doxorubicin, and Methorexate showed improved drug encapsulation and drug release properties, compared to Mitomycin-C and 5-Fluorouracil. We demonstrated the biocompatibility of the co-assembled systems, as well as their ability to intracellularly release the drugs, particularly for Epirubicin, Doxorubicin, and Methorexate. Zinc and nitrate were shown to be important in the co-assembly, coordinating with drugs and/or Cyclo-HH, thereby enabling drug-peptide as well as drug–drug interactions in successfully formed nanocarriers. The insights could be used in the future design of advanced cancer therapeutic systems with improved properties.

## Introduction

Self-assembling peptides comprise a class of highly attractive nanomaterials with promising applications in biomedicine, including the field of drug delivery,^1−5^ A reductionist approach has led to the identification of extremely short peptides, including dipeptides, capable of forming well-ordered β-sheet-rich assemblies with biological, chemical, and physical properties comparable to those of supramolecular structures formed by much larger polypeptides and proteins. In addition, the co-assembly approach has been utilized to extend the chemical space and fabricate supramolecular peptide-based architectures with improved properties.^6−8^ Nanomaterials formed by self-assembling and co-assembling peptide systems can be advantageous due to their potential biocompatibility and ability to possess tunable bioactivity; in addition, they can be designed for efficiently targeting particular sites, loading different drugs, and possessing triggered drug release at disease sites.^1,2,9,10^ Thus, self- and co-assembling peptide nanomaterials constitute a highly attractive class of drug delivery systems, for several applications including cancer delivery,^1^ allowing drug release and/or stability, in combination with reduced side effects.^1,11^

Chemotherapy is a multifaceted procedure, which includes, among others, the selection of a drug or combination of drugs to be administered.^1,12,13^ Combination chemotherapy is widely implemented in the clinic for enhanced cancer treatment^14^ due to the diversity of mechanisms involved in cancer. Combination therapy through the utilization of nanomaterial-based drug carriers requires additional investigation at both preclinical and clinical levels.^15^ Yet, the application of chemotherapy is associated with several challenging aspects, including instability in vivo, development of drug resistance, and adverse side effects due to nonspecific targeting.^16^ One emerging solution is the development of nanocarrier systems allowing for encapsulation and target-specific administration of drugs of choice. In this context, several considerations, including localization, biodistribution, biocompatibility, and efficacy of nanodrug systems in vivo, in the effort to attain precision cancer diagnosis and therapy, are important to be investigated.^15^ Loading of various therapeutic types (from small molecules to proteins^17^) was demonstrated for the delivery of cancer therapeutics using nanoparticles (i.e., polymeric micelles) in clinical trials.^18,19^ Nevertheless, it has been challenging to design nanocarriers for combinations of different cancer drugs,^16^ and only a few nanomaterial-based systems are in clinical use.^11^

A series of studies reported the computational and experimental design of cancer drug delivery material systems through the use of peptide self-assembly (reviewed in ref (1)), some of which emphasized on the importance of cyclization, and metal coordination; cyclization combined with assembly could lead to the formation of particular conformations, promote self-assembly propensity,^20^ and therefore facilitate the stabilization of particular assembled states, while metal coordination can lead to enhanced fluorescence (reviewed in ref (1)). We recently demonstrated the self- and co-assembly of cyclic dipeptides comprising natural aromatic amino acids into supramolecular nanostructures of diverse morphologies that possessed intrinsic emissions in the visible region of the electromagnetic spectrum.^21^ This process occurred through the attraction and pulling of metal ions from the solvent into the peptide environment, hence suggested to represent an “environment-switching” doping mechanism.^21^ Subsequently, we showed that the co-assembly of Cyclo-HH peptides with Zn^2+^, NO_3_^–^ and the anticancer drug Epirubicin (EPI) resulted in the formation of a nanocarrier capable of effectively delivering the drug into cancer cells while allowing in situ monitoring.^22^ In particular, we observed that the release behavior of the nanocarrier could be monitored through the variation of the fluorescent signal of Cyclo-HH in combination with Zn^2+^, demonstrating that it not only promoted the transport of EPI into HeLa cells but also could serve as a real-time optical monitor for the drug release process. Therefore, the fluorescence of the peptide nanostructures could be used to investigate the spatiotemporal distribution of the drug release process, potentially allowing the monitoring of the metabolism kinetics of the cancer drug in a certain organ or tissue.^22,23^ Computational methods were used to investigate the co-assembly properties of the formed nanocarrier, for a relatively short simulation duration, depicting the capacity of the drug to be self-encapsulated and the ability of Zn^2+^ to be less solvent-exposed and more densely packed in the presence of NO_3_^–^, providing insights into the enhanced fluorescence observed.^22^

Here we aimed to further examine the potential use of Cyclo-HH as a nanocarrier for the delivery of other cancer drugs. Extensive computational and experimental approaches examined the ability of Cyclo-HH, in combination with Zn^2+^ and NO_3_^–^, to serve as a nanocarrier through its co-assembly with EPI, and additional cancer drugs commonly used in combination therapy: Doxorubicin (DOX), Methotrexate (MTX), Mitomycin-C (MIT), 5-Fluorouracil (5FU), and Cisplatin (CIS). The drugs were chosen based on their clinical importance and therapeutic efficacy, and the fact that a portion of these drugs are used, in part, in combination therapies. For example, a combination of CIS and DOX was suggested for its increased cytotoxicity toward ovarian cancer;^14,24^ a combination of CIS, 5FU, and DOX was suggested for its efficacy against drug-resistant liver cancer;^14,25,26^ and, a combination of CIS, 5FU, and EPI was clinically tested as perioperative chemotherapy for locally advanced, respectable gastric or gastro-esophageal junction adenocarcinoma.^27,28^ Importantly, we investigated drugs with diverse structural and physicochemical properties in our effort to explore the systems’ capacity in different drug paradigms. Key properties of the systems were examined through a combination of computational and experimental methods including drug self-encapsulation, fluorescence, and the kinetics of drug release. Additionally, an extensive computational structural and biophysical analysis of the features leading to the formation of co-assembled systems formed within the simulations allowed us to obtain a fundamental understanding of the key determinants associated with successful drug nanocarriers. Our study can constitute a basis for the future design of cancer therapeutic drug delivery systems with improved properties.

## Computational Methods

### Analysis of Co-assembled Clusters within Molecular Dynamics Simulations

The structure of Cyclo-HH (Cyclo-*l*-his-d-his) was obtained from Chen et al.,^22^ and the structures of EPI, DOX, MTX, MIT, and 5FU were obtained from Pubchem (accessed on October 2022).^29^ For EPI and DOX, the structures were protonated using Arguslab (program version 4.0.1). This is in line with the fact that EPI and DOX are predominantly positively charged at neutral conditions, as the two epimer molecules p*K*_a_ lie in the range of 8.1 and 8.3.^30−33^ The structure of CIS was obtained from Yesylevskyy et al.^34^ (solvated version). Cyclo-HH and all drugs except CIS were parametrized using CGENFF^35,36^ (program version 2.5, compatible with the CGenFF version 4.6; accessed on October 2022). CIS was parametrized in accordance with the topologies of quantum dynamics performed in water solvent by Yesylevskyy et al.,^34^ which was also previously used in other studies.^37−40^

The co-assembly properties of six systems were investigated independently using explicit solvent Molecular Dynamics (MD) simulations. In each system, multiple copies of Cyclo-HH peptides were allowed to co-assemble in the presence of multiple copies of each of the six drugs shown above, and in the presence of ions and a solvent, as described below. Multicomponent assembler of CHARMM-GUI (accessed on October 2022),^41−44^ was used to build each of the six systems comprising 48 copies of Cyclo-HH peptides, 12 copies of each drug, and 48 Zn^2+^, solvated in an 83 Å cubic periodic boundary conditions 95:5 IPA/DMF box, in accordance with the experimental analyses (see below); parameters for NO_3_^–^ were obtained from ref (45), while parameters for the solvent molecules and Zn^2+^^46^ were provided by CHARMM-GUI.^41 −44^ It is worth noting that the simulated concentration was higher compared to experiments, as a means to computationally enhance the co-assembly process,^7,22,47^ which in this case was experimentally enhanced by heating and subsequent equilibration of the systems at room temperature (see below). The system was neutralized with the addition of 96 NO_3_^–^, with the exception of MTX in which 72 NO_3_^–^ were added only due to the −2 net charge of MTX. For systems comprising EPI and DOX, 12 Cl^–^ ions were additionally added to maintain neutrality due to the +1 net charge of EPI and DOX. Peptides and drugs were automatically built with random positions within space, without any user-predefined positions, and subsequently, a Monte Carlo placing of ions was employed, using CHARMM-GUI.^41−44^ After the systems were prepared using all steps provided by the multicomponent assembler, a short equilibration *NVT* simulation was performed, followed by a long *NPT* production simulation in OpenMM,^42,43^ using the default parameters and setup provided by CHARMM-GUI.^41−44^ Finally, Lennard-Jones interactions were scaled to zero at a distance of 12 Å, a time step of 1 and 2 ps was used in the *NVT* and *NPT* (1 atm) MD simulation runs, respectively, and the temperature was controlled by a Langevin thermostat at 300 K using a friction coefficient of 1 ps^–1^. 300 K was chosen in accordance with the room temperature at which the systems are allowed to equilibrate after heating in the experimental analyses. The total simulation duration for each system was 1 μs. Snapshots were saved and analyzed every 1 ns. Each of the six systems was built and simulated in triplicates, i.e., 3 μs in total were produced and analyzed for each of the six drugs under investigation.

### Structural and Energetic Analysis of the Simulated Systems

Visual inspection of the runs showed that clusters were formed within the simulated trajectories by co-assembly of Cyclo-HH, Zn^2+^, NO_3_^–^, and drugs. In-house FORTRAN programs were developed to identify multicomponent clusters, comprising different components of Cyclo-HH peptides, drugs, and ions Zn^2+^ and NO_3_^–^. To detect such clusters, a 3.5 Å distance cutoff was set to identify interacting atom pairs of different Cyclo-HH peptides, drugs, and ions. Thus, if the distance between any atom pair in different peptides, drugs, and ions was below the cutoff value, the particular peptides, drugs, or ions were considered to be interacting with each other in the particular snapshot. Overall, all possible combinations of interacting pairs of peptides, drugs, and ions, that can be part of a cluster were exhaustively searched for; an analogous definition was used in ref (22). It is important to note that, following this criterion, if two peptides, drugs, or ions interact with each other, and concurrently with another peptide, drug, or ion (e.g., if two Cyclo-HH peptides interact with each other, and also their interaction is mediated by a Zn^2+^), all (three in this case) interactions are explicitly considered. Nearest neighbor interactions were not exclusively considered (i.e., no prioritization was imposed), allowing us to identify and highlight the important role of (all) direct interactions between peptides and/or drugs, as well as importantly how Zn^2+^ and/or NO_3_^–^ mediate their interactions. Upon clusters’ detection, their size was equal to the sum of all interacting peptides, drugs, and ions. Notably, the periodic boundary conditions were considered in the identification of interacting atm pairs, and therefore, in the cluster detection and representation as well.

The programs detected the formation of clusters ranging in size, with the largest cluster identified comprising more than 140 peptides, ions, and drugs. Due to the fact that the current setup and simulated conditions advantageously allowed the formation of clusters with a large number of peptides, ions, and/or drugs in nearly all systems under investigation (all six drugs), the computational analysis focused on larger clusters (i.e., more than 30 peptides, ions, and drugs), representing co-assembled clusters of higher complexity, which could more likely be related to the assemblies observed experimentally. It is worth noting that per snapshot analyzed two or more clusters could be independently detected and analyzed. The number of clusters as a function of the clusters’ size was determined, as well as the probability of a drug being encapsulated in a cluster, along with the percentage of drug encapsulation as a function of the clusters’ size. Additional details about these calculations are provided in the Supporting Information Methods. The analysis focused on clusters incorporating drugs. Nevertheless, clusters without any drug, which were mostly formed in the systems with drugs MIT, 5FU, and predominantly CIS (see below), were collected and used primarily for comparison purposes.

A series of structural properties of the formed clusters were extracted and analyzed as a function of clusters’ size: (i) the percentage composition per component in a cluster; (ii) the probability of each Cyclo-HH peptide and drug to interact with other peptides, drugs and ions (excluding repulsive pairs Zn^2+^–Zn^2+^, NO_3_^–^ - NO_3_^–^); (iii) the percentage ratio of solvent accessible surface area divided by the total surface area per component (where solvent accessible surface area calculations were performed using Wordom^48−50^ and the probe radius of IPA was taken from ref (51−52) (see Supporting Information)); (v) the radius of gyration (Å) of the cluster (using Wordom^49−50^); (vi) the ratio of the drugs divided by the Cyclo-HH peptides; (vii) the ratio of Zn^2+^ divided by the Cyclo-HH peptides; and (vii) the probability of a peptide, drug, and ion (Zn^2+^ or NO_3_^–^) to mediate interactions with Cyclo-HH peptides or drugs. The ensemble of features of all components for (iii) and (vii) were also provided as input to a multiclass Support Vector Machine (SVM) model using binary learners in Matlab to uncover which combinations of these two properties mostly differentiate between clusters of EPI, DOX (defined as a first class), MTX (defined as a second class), and MIT as well as 5FU (defined as a third class). Analytical details about the definition and calculation of all features as well as the development of the SVM model are provided in the Supporting Information Methods. CIS was excluded from the class definitions due to its significantly lower capacity to be encapsulated.

All drugs and Cyclo-HH were decomposed into chemical groups (Figure S1), analogously to our previous study,^53^ and the normalized probability of different drug and peptide chemical groups to interact with Zn^2+^ and NO_3_^–^; or other drugs’ and peptides’ chemical groups were additionally analyzed. In this case, an interaction was defined between two chemical groups of the peptide or the drug rather than between the two molecules (peptides, drugs) in total (corresponding to all of the above calculations). Finally, the average free energy for each drug and peptide (as an individual molecule) to be associated with a preformed co-assembled cluster was calculated, with all other peptides, drugs, and ions at present, for the 20 highest complexity structures formed within the simulations of each system, followed by energy minimization in CHARMM,^54^ and energy calculations using Autodock4Zn.^48^ The aforementioned energy analysis was performed for all drug systems individually, except CIS which according to results (see below) showed a significantly lower propensity to co-assemble with the rest of the system. It is important to note that Autock4Zn was used for energy calculations of the minimized energy simulated snapshots and not for docking (or redocking of drugs) within the preformed co-assembled clusters. Additional details about the interactions involving molecular decomposition into chemical groups, as well as on association energy calculations, are provided in Supporting Information Methods.

## Experimental Methods

### Chemicals

The Cyclo-HH peptide was purchased from GL Biochem (Shanghai, China). Zinc nitrate (Zn(NO_3_)_2_), dimethylformamide (DMF), dimethyl sulfoxide (DMSO), isopropanol, doxorubicin, and ethanol were purchased from Sigma-Aldrich (Rehovot, Israel). EPI hydrochloride was purchased from Glentham Life Science and doxorubicin hydrochloride (DOX) from Sigma-Aldrich (Rehovot, Israel). Additionally, MTX, MIT, 5FU, and CIS were purchased from Holland Moran, Israel. All materials were used as received without any further purification. Highly pure deionized water was processed using a Millipore purification system (Darmstadt, Germany) with a minimum resistivity of 18.2 MΩ cm.

### Co-Assembly of Cyclo-HH, Zn^2+^, and NO_3_^–^ with Cancer Drugs

The co-assembly of Cyclo-HH, Zn^2+^, and NO_3_^–^ with or without cancer drugs was prepared following the literature protocol.^22^ The co-assembly was independently performed using the six different drugs, EPI, DOX, MTX, MIT, 5FU, or CIS, with slight modifications in terms of solvent (DMF/isopropanol), reaction time, and temperature. A fresh stock solution of Cyclo-HH was prepared by dissolving the peptide in 5% (v/v) DMF/isopropanol at a concentration of 4 Cyclo-HH peptide mole, 4 Zn(NO_3_)_2_ mole, and 1 mol ratio of each cancer drug (EPI, DOX, MTX, MIT, 5FU, or CIS) under water bath sonication, followed by incubation at 80 °C for 2 h.^22,23^ In order to remove nonencapsulated excess drugs, unbound ions (Zn^2+^/NO_3_^–^), or unreacted salts (Zn(NO_3_)_2_), the obtained suspension was centrifuged at 14,000 rpm for 30 min, and the precipitate was washed three times with deionized water. The obtained materials were lyophilized to obtain a solid powder.

### Transmission Electron Microscopy

Nanostructures of Cyclo-HH, Zn^2+^, and NO_3_^–^, individually co-assembled with EPI, DOX, MTX, MIT, 5FU, or CIS after 2 h of chemical reactions were added to a copper grid (400 mesh) coated with a thin carbon film for 2 min. In the following steps, the excess solution was removed, and the grid was washed three times with DI water. The TEM images were obtained using a JEM-1400Plus electron microscope operating at 80 kV. Images were analyzed using the ImageJ software. In order to ensure accuracy, triple measurements were performed and averaged for each co-assembly.

### Fluorescence Spectroscopy

Samples of co-assembled nanostructures synthesized as outlined above dispersion/solution were pipetted into a quartz cuvette with a path length of 1.0 cm, and the spectrum was collected using a FluoroMax-4 Spectrofluorometer (Horiba Jobin Yvon, Kyoto, Japan) at ambient temperature. Excitation and emission wavelengths were set at 300–500 nm and 350–750 nm, respectively, with a slit of 2 nm.

### Drug Release Profiles

We conducted a detailed analysis of the release kinetics for all six drugs formed by the co-assembly of Cyclo-HH, Zn^2+^, and NO_3_^–^ with each of the drugs using UV–visible spectroscopy. An in vitro drug release profile analysis was performed on the co-assembled nanostructures of Cyclo-HH, Zn^2+^, and NO_3_^–^ with EPI, DOX, MTX, MIT, 5FU, or CIS samples (5.00 μg/mL), in comparison to co-assembled nanostructures in the absence of drugs, as well as each pristine drug (0.7 μg/mL) with dialysis in 25 mL PBS buffer (pH 7.4) or acetate buffer (pH 6.0) using an Agilent Cary 100 UV–visible spectrophotometer equipped with a quartz cuvette of 1.0 cm path length. The dialysis was performed in an incubator shaker at 37 °C. Under this condition, it was assumed that drug release would begin at normal human body temperature (37 °C) and different buffers (pH 7.4 or 6.0) similar to body fluids. At predetermined intervals, aliquots (200 μL) were removed from the release reservoir solution at various time points for characterization via UV–vis spectrophotometry. The quantification of the released drug concentration was achieved by measuring the absorption at specific wavelengths, with 490 nm used for EPI and DOX, based on calibration curves established under comparable conditions (pH 6.0 and 7.4). The release profiles for MTX, MIT, 5FU, and CPT were similarly assessed at their respective characteristic absorption wavelengths of 302, 363, 265, and 312 nm.

### Cell Viability Analysis

For cytotoxicity analysis, 1 × 10^6^ HeLa cells/mL cells were cultured in 96-well tissue microplates (100 μL per well) and allowed to adhere overnight at 37 °C. Co-assembled nanostructures of Cyclo-HH, Zn^2+^, and NO_3_^–^ with or without EPI, DOX, MTX, MIT, 5FU, or CIS were added to the cell growth medium at concentrations of 1, 2, and 4 μg/mL. One-half of each plate was seeded with cells, while the other half served as a blank control. As a negative control, a medium without nanostructures was used. A cell viability assay was performed using 3-(4,5-dimethylthiazolyl-2)-2, 5-diphenyltetrazolium bromide according to the manufacturer’s instructions following a 24 h incubation at 37 °C. Briefly, after 24 h incubation at 37 °C, 10 μL of 5 mg/mL MTT reagent dissolved in PBS was added to each of the 96 wells, followed by a 4 h incubation at 37 °C. The wells were then filled with 100 μL of extraction buffer (100% DMSO) and incubated at 37 °C in the dark for 30 min. Lastly, absorbance intensity was measured using a multiplate reader at 570 nm and background subtraction was performed at 680 nm.

### Live Cell Imaging

Images of live HeLa cells with co-assembled nanostructures of Cyclo-HH, Zn^2+^, and NO_3_^–^ with the EPI, DOX, MTX, MIT, 5FU, or CIS were obtained using confocal microscopy. In brief, the HeLa cells were grown in glass bottom dishes until 70 to 80% confluence. Afterward, the cells were cultured with media containing drug-co-assembled nanostructures at a concentration of 4 μg/mL for different periods of time. Then, the cells were washed twice with PBS. Imaging was performed by using a SP8 inverted confocal microscope (Leica Microsystems, Wetzlar, Germany). The ranges of excitation and emission were: EPI or DOX, MTX, MIT, 5FU, or CIS λ_ext_ = 488 nm, λ_em_ = 510–590 nm; and for Hoechst live cell nucleus staining dye, λ_ext_ = 405 nm, λ_em_ = 420–500 nm. An additional barrier filter was used in order to block light emission above 590 nm. The emission light was separated by a dichroic mirror (555 nm), and the two fluorescent lights were filtered by two bandpass filters (500–550 and 540–690 nm).

## Results

### Morphological and Structural Studies

**Figure 1:**
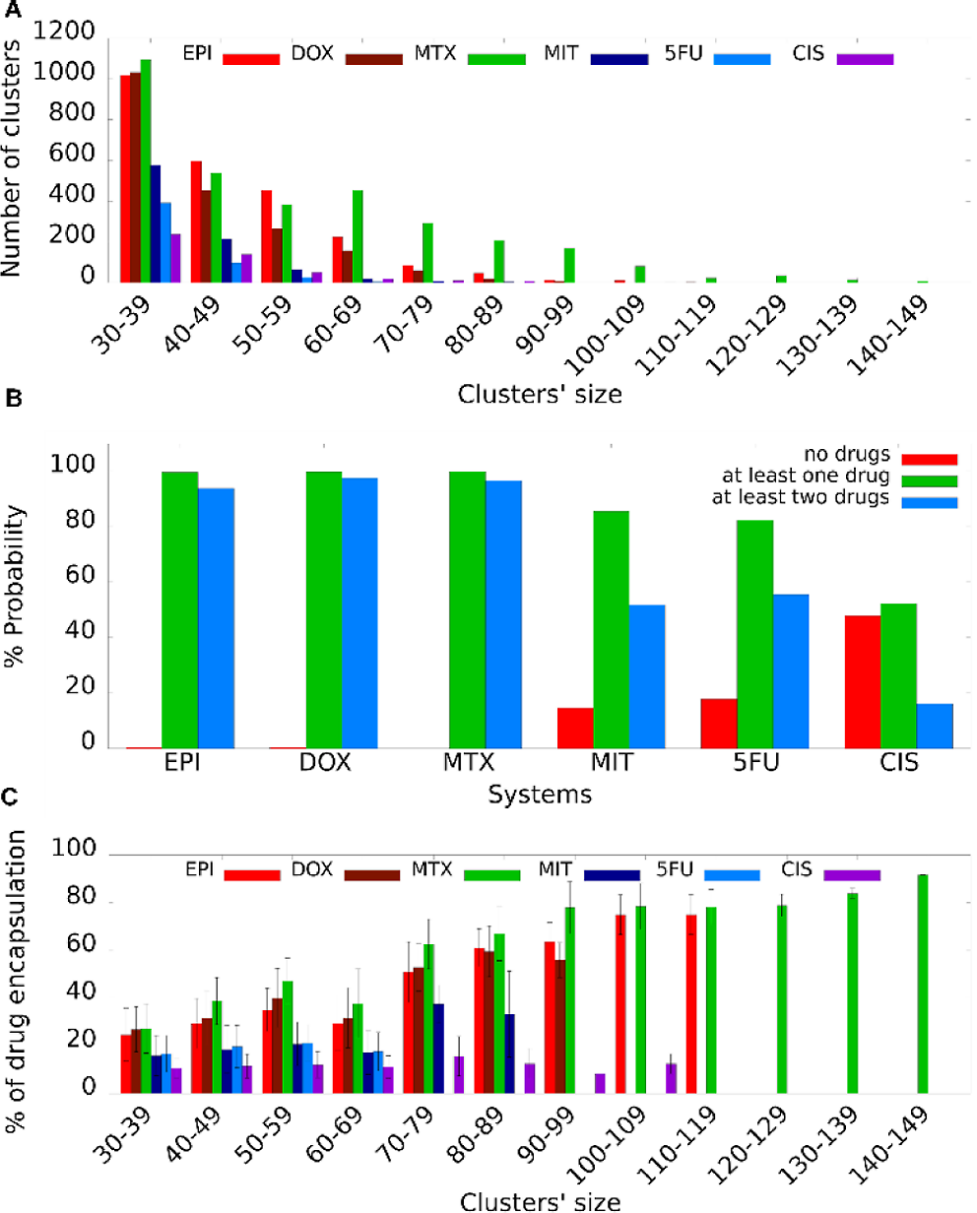
(A) The number of clusters as a function of the clusters’ size per system; EPI (red), DOX (maroon), MTX (green), MIT (dark blue), 5FU (light blue), or CIS (purple). (B) The percentage probability of (i) no drug encapsulated (red), (ii) at least one drug encapsulated (green), and (iii) at least two drugs encapsulated (blue) per simulated system. (C) The percentage of drug encapsulation as a function of the cluster’s size for clusters with EPI (red), DOX (maroon), MTX (green), MIT (dark blue), 5FU (light blue), and CIS (purple).

**Figure 2:**
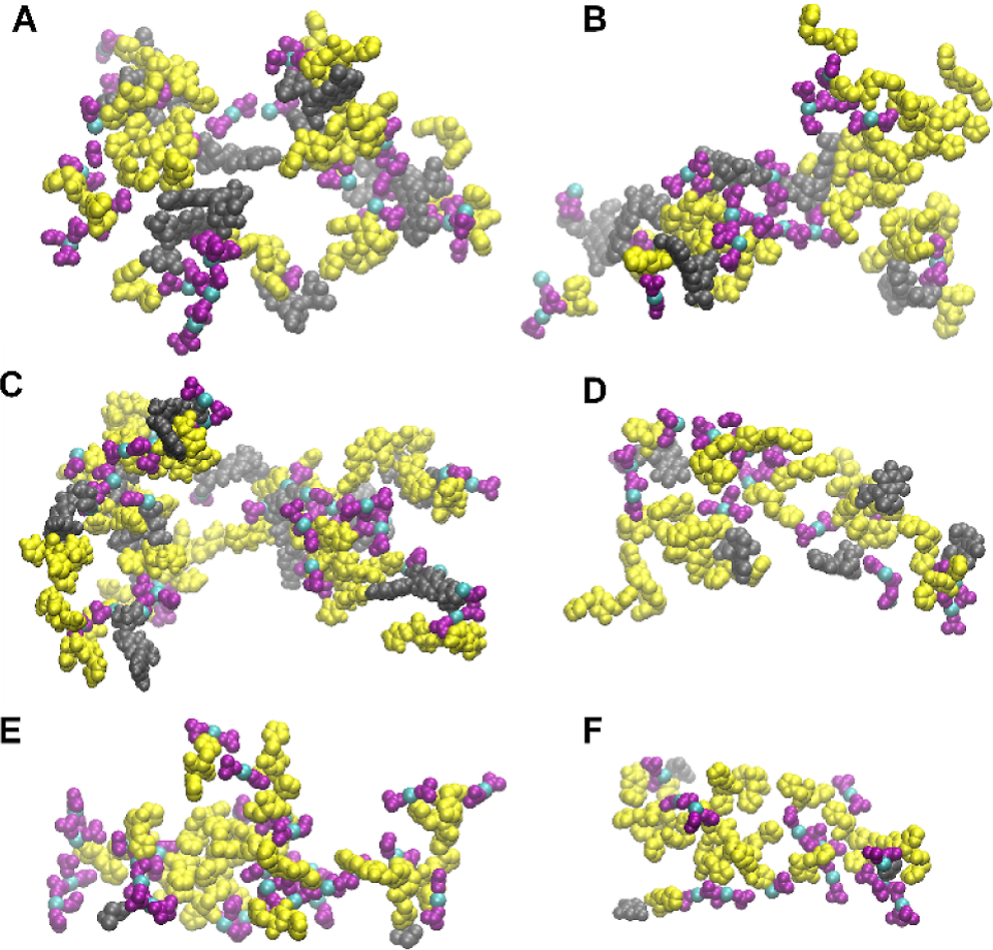
Molecular graphics images of the highest complexity clusters formed in MD simulations for each system: (A) EPI, (B) DOX, (C) MTX, (D) MIT, (E) 5FU, and (F) CIS. The Cyclo-HH peptides are shown in yellow VDW representation, the drugs in gray VDW representation, Zn^2+^ in cyan VDW representation, and NO_3_^–^ in purple VDW representation.

Within the simulations of all six systems, co-assembled structures containing a large number of peptides, ions, and drugs were formed. A larger number of clusters and clusters of larger size were formed for EPI, DOX, and MTX in comparison to MIT, 5FU, and CIS (Figure 1A), independent of the presence of a drug in the specific cluster. The simulations depicted a significant trend for co-assembly for the systems encapsulating EPI, DOX, and MTX a reduced trend for MIT, 5FU, and even less for CIS. With the exception of very few cases, all clusters in the simulated systems of EPI, DOX, and MTX contained at least one drug molecule and in the vast majority of cases they contained two drug molecules or more. In the case of MIT and 5FU, approximately 20% of the clusters did not contain any drug, whereas in the case of CIS, this percentage was approximately 50% (Figure 1B). Thus, the probability of drug encapsulation in the simulated systems was nearly thorough for EPI, DOX, and MTX, followed by MIT, 5FU, and less for CIS. Thus, the larger number of clusters and clusters of large size within the simulated systems of EPI, DOX and MTX, should be considered an outcome of a higher propensity of these drugs to be encapsulated and be part of the formed cluster. Examples of high-complexity clusters formed per simulated system are depicted in Figure 2, and their coordinates, except for CIS, are provided as Supporting Information data in PDB format.

Within the clusters containing drugs in the simulations, the percentage of encapsulated drug molecules out of all drug molecules available per system was overall higher in clusters with EPI, DOX, and MTX in comparison with MIT and 5FU, and even less in CIS (Figure 1C). Interestingly, the percentage overall seemed to increase for clusters of larger size for the cases and systems that such clusters were detected in the simulations. Particularly for clusters comprising EPI, DOX, and MTX, at least 50% or more of the drugs in the system were part of clusters formed (i.e., encapsulated) for clusters containing at least 70 or more peptides, drugs, and ions (Figure 1C). The higher percentage of drug encapsulation in clusters of EPI, DOX, and MTX is demonstrated by the higher number of drug molecules in the representative clusters (Figure 2A–C) (gray VDW representation) compared to the clusters of MIT, 5FU, and CIS (Figure 2D–F).

In all simulated systems, NO_3_^–^ was the main component of each cluster, followed by Cyclo-HH and Zn^2+^, and NO_3_^–^ were overall approximately twice as many as Zn^2+^, with the exception of the clusters with MTX, in which the Zn^2+^ component was higher than all other systems and was neither lower than Cyclo-HH nor half of NO_3_^–^ (Figure S2). This is attributed to the capacity of Zn^2+^ to coordinate with MTX, in addition to Cyclo-HH. The percentage composition of drugs did not exceed ∼10% for clusters in all systems, and this value was almost uniform in the clusters of MTX, EPI, and DOX. The corresponding percentage was lower in the clustered systems of MIT and 5FU, and even lower for CIS (Figure S2). Also, comparing all clusters with drugs, except MTX, the percentage composition of Cyclo-HH, Zn^2+^, and NO_3_^–^ was overall uniform and slightly smaller compared to clusters without any drug, indicating that the presence of drugs did not alter the relative proportion of elements in the cluster (Figure S2).

**Figure 3:**
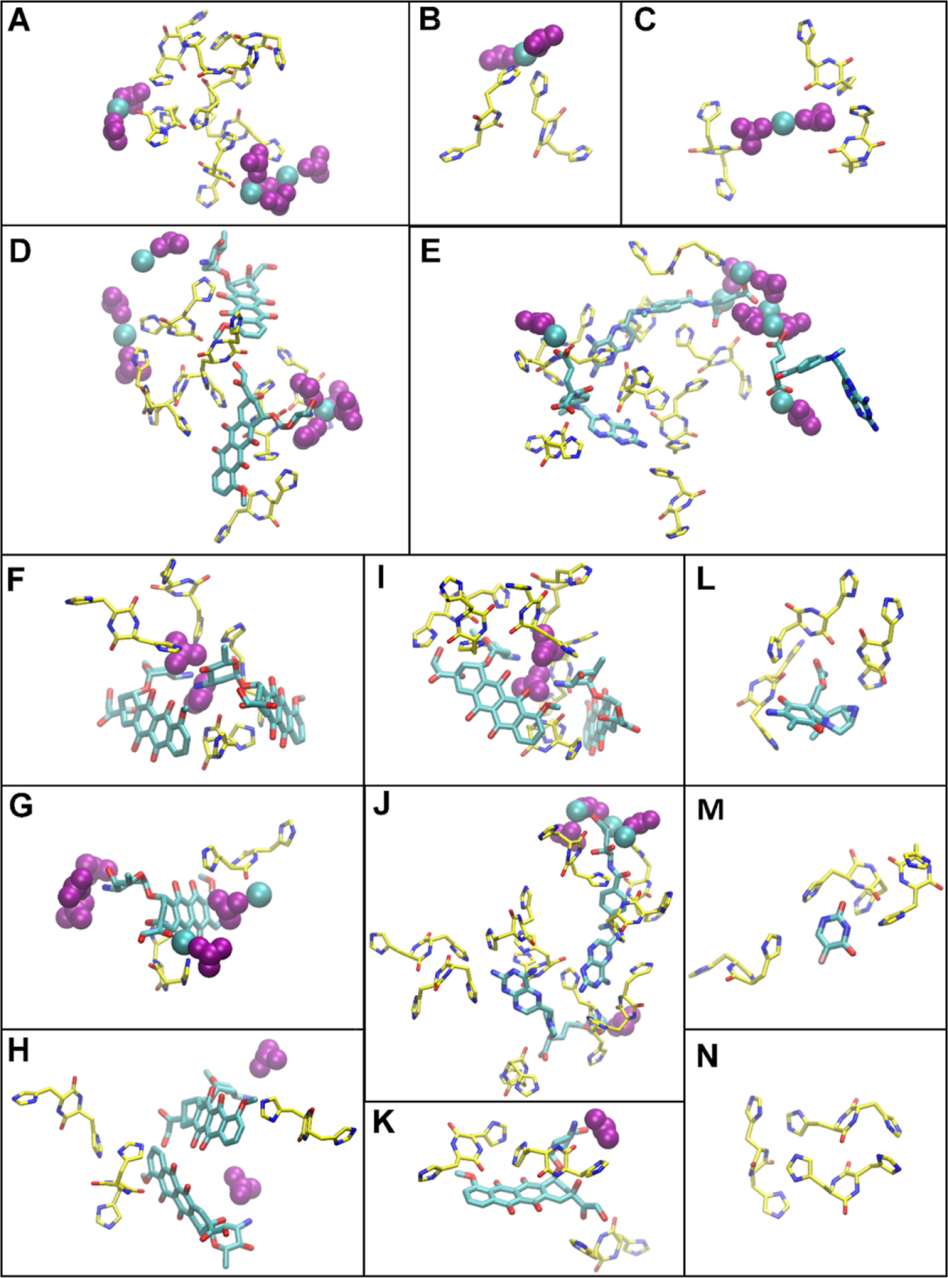
Molecular graphics images of the peptides, drugs, and ions interacting within the clusters. The Cyclo-HH peptides and the drugs are shown in licorice yellow and cyan representation; the Zn^2+^ and NO_3_^–^ are shown in cyan and purple VDW representation, respectively. Panels (A–C) and (N) present interactions between Cyclo-HH and/or ions; panels (D–I) and (K), present interactions involving EPI or DOX; panels (E–J) present interactions involving MTX; panel (L) presents interactions involving MIT; panel (M) presents interactions involving 5FU.

The analysis presented above shows that drugs participated in the co-assembly process and that different drugs, with diverse structural and physicochemical properties, had different propensities to be co-assembled and encapsulated within clusters containing Cyclo-HH, Zn^2+^, and NO_3_^–^. Overall, within the clusters observed in the simulations, encompassing drugs or not, the peptides had a strong tendency to interact with each other, followed by NO_3_^–^ and Zn^2+^ in decreasing order (Figure S3). Particularly, we observed that a Cyclo-HH–Cyclo-HH interaction could be direct (Figure 3A) or mediated by Zn^2+^ and/or NO_3_^–^ (alone or as a pair; Figure 3B,C). Notably, within the drug-encompassing clusters, Cyclo-HH-drug interactions were approximately half of the Cyclo-HH–Cyclo-HH interactions for EPI, DOX, and MTX, less than a quarter of Cyclo-HH–Cyclo-HH interactions for MIT, and 5FU, and substantially less for CIS (Figure S3). This is in line with the corresponding drug encapsulation capacities of each system, and reflects that EPI, DOX, and MTX showed higher tendencies to interact with Cyclo-HH, either directly or mediated by ions (see above), followed by MIT and 5FU. Hence, a higher probability of a direct or indirect interaction between a drug and Cyclo-HH within the co-assemblies resulted in significantly improved drug encapsulation.

In all simulated systems incorporating drugs, the percentage of the solvent accessible surface area over the total solvent accessible surface area, which was calculated for each peptide, drug and ion in a cluster, and averaged for the four different components (peptides, drugs, Zn^2+^, and NO_3_^–^), was approximately equal for the drugs and peptides, and higher than that of the two less abundant components, NO_3_^–^ and Zn^2+^ (Figure S4). Importantly, overall, NO_3_^–^ was more exposed than Zn^2+^ (Figure S4, Figure 2). Zn^2+^ was slightly more buried and NO_3_^–^ slightly more exposed in the clusters comprising MTX, which can be related to the prevalent tendency of Zn^2+^ to coordinate with the drug (toward the interior) in this system compared to others. Similarly, Zn^2+^ was slightly more buried and NO_3_^–^ slightly more exposed in the systems comprising 5FU and MIT compared to EPI and DOX which can be related to the fact that the latter drugs prevalently interacted with NO_3_^–^, and in such interactions, the Zn^2+^ coordinated with NO_3_^–^ were more exposed. Additionally, while the degree of exposure of drugs was relatively high and varied, in the clusters containing 5FU, the solvent accessibility of the drug was smaller compared to other systems (Figure S4), potentially attributed to the overall smaller size of the drug along with its capacity to be encapsulated (Figures 1B,C and S2). Overall, all components in clusters of all systems within the simulations were assembled in mixed patterns, with drugs distributed toward the interior or exterior of the mixed assemblies (Figure 2). This leads to a different degree of exposure for all components which is more related to how the peptides, drugs, and ions interact with each other, and not necessarily to their position in the cluster. Primarily, the general trend of the above-described degree of exposure for Cyclo-HH, NO_3_^–^ and Zn^2+^ in the clusters encapsulating drugs was similar to the trend of the clusters without any drugs, suggesting that the drugs do not have a significant impact on this aspect, at least under the particular conditions investigated here. Furthermore, the size of the clusters, indirectly depicted by the radius of gyration, increased as a function of the number of peptides, drugs, and ions in the cluster (Figure S5). However, it is worth noting that the larger the drug and the larger the number of drugs in a cluster, the larger the cluster formed was (Figure S5).

While the initial ratio in the simulation setup of drug to Cyclo-HH peptides was uniformly equal to 1:4 (=0.25) in the simulated systems, the corresponding ratio ended up being larger than ∼0.30 in the clusters of co-assembled systems comprising EPI, DOX, MTX, and smaller than ∼0.20 in the clusters of co-assembled systems comprising MIT and 5FU, and even lower for CIS (Figure S6), in line with the aforementioned analysis. The ratio of Zn^2+^ to Cyclo-HH in the clusters of all systems (except CIS) was within the approximate range of ∼0.80 to 1.00, and was overall slightly higher in clusters of MTX due to the drug’s ability to coordinate with Zn^2+^. The ratio of Zn^2+^ to Cyclo-HH peptides was also higher in larger clusters of CIS (Figure S7), reflecting the low number of drugs in that system. In fact, the ratio was similar to those in cases where no drugs were present.

The degree of drug encapsulation differentiated the clusters of CIS from clusters of all other drug systems as well as distinguished between EPI, DOX, and MTX (with a higher degree of drug encapsulation), with MIT and 5FU (with a lower degree of drug encapsulation). The different degree of drug encapsulation was related with the drugs’ ability to co-assemble within the cluster (interacting with other drugs or Cyclo-HH, Zn^2+^, and NO_3_^–^) in the simulations. Especially, in clusters encapsulating EPI, DOX, and MTX, the drugs had a higher tendency to interact with Cyclo-HH and with each other as well, compared to MIT and 5FU; in contrast, in CIS such interactions were significantly reduced (Figure S8). In line with this, the probability of PDP, PDD, DDD, PPD, and DPD interactions, where the middle molecule, either peptide (P) or drug (D), mediates interactions with two other peptides (P) and or drugs (D), was higher or significantly higher in the clusters of EPI, DOX and MTX compared to the clusters with MIT and 5FU (Figures S9 and S10). The probability of PPP was higher in clusters with MIT, and 5FU (and substantially higher in clusters with CIS), which can be attributed to the fact that it is less probable for these drugs to form PDP interactions (Figures S9 and S10). A network of multiple PPP interactions is shown in Figure 3A, where the peptide interactions were not mediated by ions, while networks containing peptide-drug interactions are shown in Figure 3D,E. In particular, in Figure 3D, PPDPDPP and PPDDPP patterns encountered within clusters of EPI are presented, demonstrating the capacity of the drug to directly interact with peptides. Similarly, a network of interacting drugs and peptides, without the mediation of any ion, within the clusters of MTX is presented in Figure 3E.

The different tendencies of the investigated drugs to coordinate with Zn^2+^ and NO_3_^–^ played a key role in drug encapsulation, as observed within the simulations. The probability of EPI, DOX, and MTX to coordinate with NO_3_^–^ and Zn^2+^ respectively, was evidently higher than MIT and 5FU (Figure S8). Hence, the difference between the clusters of EPI, DOX, and MTX (high degree of drug encapsulation) with the clusters of MIT and 5FU (low degree of drug encapsulation) appears to constitute a synergism of the above in conjunction to the ability of EPI and DOX to coordinate NO_3_^–^ and to the ability of MTX to coordinate with Zn^2+^ (Figure S8). This synergism, given the ability of Zn^2+^ and NO_3_^–^ to interact with Cyclo-HH as well (Figure S3), seems to be the key contributing factor for enhanced drug encapsulation. Hence, ions enhanced the drug–drug and drug-peptide interactions in the case of EPI, DOX, and MTX, which is shown by the increased probability of NO_3_^–^ to mediate drugs and peptides interactions in the EPI, DOX systems and of Zn^2+^ in the case of MTX (Figures S11 and S12). Representative examples of NO_3_^–^-mediated interactions between two drugs (DND), between two peptides (PNP) and between a peptide and a drug (DNP) are shown in Figure 3F in a cluster of EPI, while a representative example of Zn^2+^ mediated interactions between two drugs (DZD) in a cluster of MTX is shown in Figure 3E. In such interactions, charge neutrality was maintained by coordination of Zn^2+^ and NO_3_^–^ forming “bridges” mediating the interactions (Figure 5E).

An SVM model (Table S1) enabled us to clarify how the degree of solvent accessibility (Figure S4) in combination with mediated interactions (Figures S9–S12) differentiates between clusters of EPI, DOX (first class), MTX (second class), and MIT and 5FU (third class). Particular mediated interactions are key in differentiating between the three classes: (i) DZD and PZD interactions were significantly prevalent in the system comprising MTX due to the coordination of the drug with Zn^2+^, and they are unique to this system. Such interactions were the key building blocks assembling drugs and peptides in the MTX system (Figure S9–S12); (ii) DND interactions were significantly prevalent in systems comprising EPI and DOX due to the coordination of the drug with NO_3_^–^, as well as in systems comprising MTX due to the partial coordination directly with NO_3_^–^ or indirectly through Zn^2+^-NO_3_^–^ interactions; DND interactions differentiate these systems to the 5FU and MIT; (iii) PZP interactions, despite being low in probability (Figure S11), were slightly more probable in systems with MTX compared to EPI and DOX, followed by 5FU and MIT differentiating the first to second, and second to third classes of systems; (iv) PND interactions were significantly prevalent in systems comprising EPI and DOX due to the coordination of the drug with NO_3_^–^. (i–iv) in conjunction with particular metrics on the degree of solvent accessibility for different components show how these features, in combination, give rise to some distinctive "in part" behavior of the three classes (Figure S13).

The analysis above showed the key role of interactions between Cyclo-HH and drugs with ions within the clusters in the simulations. Both Zn^2+^ and NO_3_^–^ possessed nearly equal probability to be in proximity to both the imidazole rings and the central cyclic ring (Figures S14 and S15), which can be attributed to the fact that Zn^2+^ coordinates with the nitrogen of the imidazole rings and with the oxygens of the cyclic ring (Figure 3A), as well as to the fact that NO_3_^–^ interacted either directly with Cyclo-HH via hydrogen bonds with polar groups in the imidazole or cyclic rings (Figure 3C) and/or indirectly through the oppositely charged interactions with Zn^2+^ bound to Cyclo-HH. Overall, these led to a relatively high probability of Cyclo-HH-NO_3_^–^ interactions. The most prevalent interactions between drugs and ions, depicted in Figures S16 and S17, comprise oppositely charged attractions between the positively charged groups of EPI and DOX with NO_3_^–^ (Figure 3F), and the negatively charged group of MTX with Zn^2+^ (Figure 3E). In addition, Zn^2+^ coordinated with particular polar groups of EPI, DOX (Figure 3G), MIT, and 5FU (Figure S16). These interactions facilitated NO_3_^–^ to be in proximity to the charged group of MTX (Figure 3E), and particular polar groups of EPI, DOX, MIT, and 5FU (Figure S17). Τhe previous analysis also showed that both drug–drug and drug-peptide interactions are more prevalent in clusters comprising EPI, DOX, and MTX in comparison to the other drugs (Figure S8). Visual inspection of the twenty-highest complexity clusters for both drug–drug and drug-peptide interactions revealed that in the case of EPI and DOX, the drugs interacted mostly either through their hydrophobic groups (Figure 3H) or through NO_3_^–^, which mediated the interactions between the polar atoms of hydrophobic groups and the charged group (Figure 3I). In clusters incorporating MTX, drug–drug interactions occurred primarily either directly between the uncharged groups of the drug (Figure 3J) or indirectly between the charged groups facilitated by networks of ions (Figure 3E). In contrast, no particular modes of drug–drug interactions could be observed for MIT and 5FU, which can be related to their lower tendency to interact with each other (Figure S8). For CIS, the interactions between drugs were almost absent. As for the drug-peptide interactions in clusters of EPI, DOX, and MTX, all groups of these drugs could interact with the imidazole rings or cyclic ring of Cyclo-HH either directly (Figure 3K,E respectively) or indirectly, through ion-mediated interactions (Figure 3F,E respectively). In contrast, for MIT and 5FU, the drugs interacted with the imidazole ring and cyclic ring of the Cyclo-HH mostly directly (Figure 3L,M respectively). This reconfirmed the key role of ions in the clusters of EPI, DOX, and MTX, in which ions enhance (or mediate) drug–drug and drug-peptide interactions. This is in also line with the high probability of the mediated DND and PND interactions for the clusters encapsulating EPI and DOX compared to the other tested (Figure S12), and with the high probability of the mediated DZD and PZD interactions for the clusters of MTX (Figure S11). Peptide–peptide interactions were also generally facilitated through networks of hydrogen bonds (Figure 3N).

**Figure 4:**
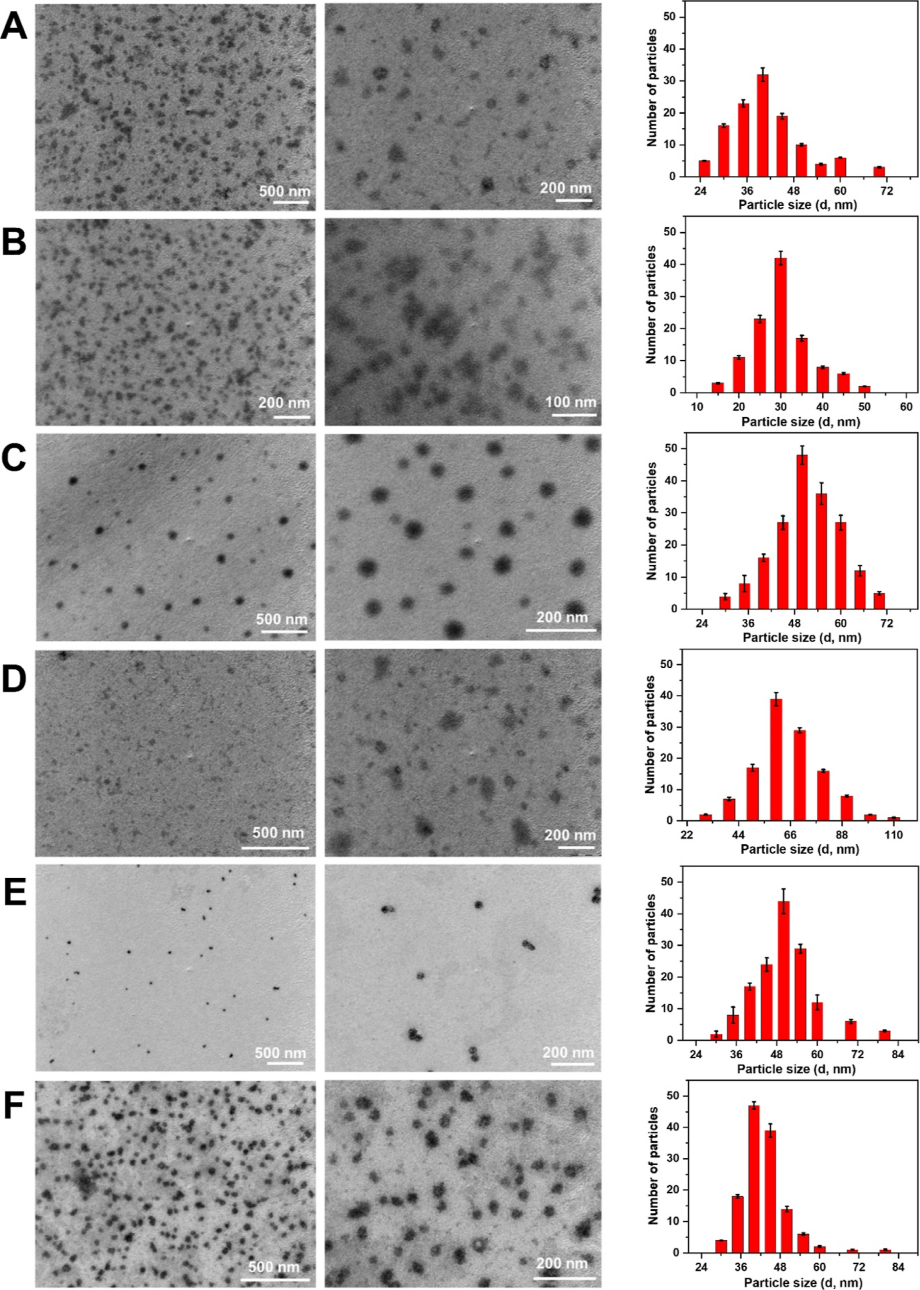
TEM images of nanostructures formed by the co-assembly of Cyclo-HH, Zn^2+^, and NO_3_^–^ with (A) EPI, (B) DOX, (C) MTX, (D) MIT, (E) 5FU, and (F) CIS. Particle size distributions based on the TEM images are shown on the right.

TEM imaging of the nanostructures formed by co-assembly of Cyclo-HH, Zn^2+^, NO_3_^–^, and EPI, DOX, MTX, MIT, 5FU, or CIS was performed to reveal their morphology. It is important to note that on the basis of TEM analysis and materials yield, it is unclear whether each of the drugs and to what extent they co-assembled within the nanostructures. The TEM images showed the average diameters of the nanostructures formed by co-assembly of Cyclo-HH, Zn^2+^, NO_3_^–^, and EPI, DOX, MTX, MIT, 5FU, or CIS to be ∼40, ∼30, 50, ∼60, ∼50, and ∼40 nm, respectively (Figure 4), demonstrating that the presence of drugs during the co-assembly could influence the nanostructures’ diameter. The variation in the size of the nanoparticles obtained from the co-assembly of Cyclo-HH, Zn^2+^, NO_3_^–^ and EPI, DOX, MTX, MIT, 5FU, or CIS could be due to the distinct co-assembling properties Cyclo-HH, Zn^2+^, NO_3_^–^ with different drugs. As for EPI, similar results were obtained in our previous study of the Cyclo-HH–Zn^2+^, NO_3_^–^, and EPI nanostructure formed by co-assembly in which we verified that EPI co-assembled with Cyclo-HH, Zn^2+^, and NO_3_^–^.^22^ The material yield of the synthesized product was slightly lower in MIT, and CIS compared to EPI, MTX, and DOX. In the case of 5FU, a slight reduction in yield was observed. The product yield was calculated according to the weight of the final product obtained for each synthesis in relation to the weight of the precursor (starting material).

**Figure 5:**
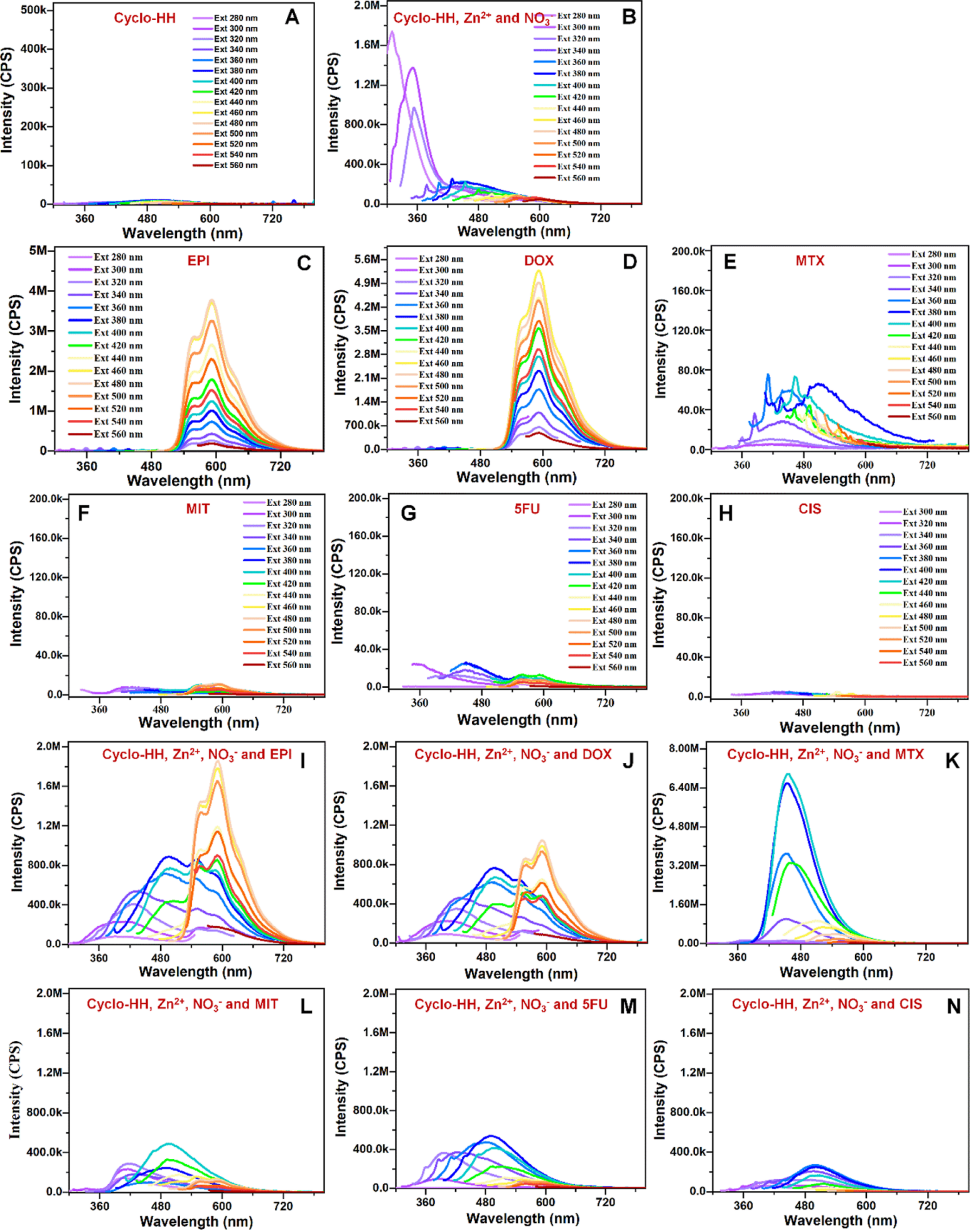
Fluorescence analysis of the systems with (A) Cyclo-HH, (B) Cyclo-HH, Zn^2+^, NO_3_^–^ nanostructures, (C) pristine EPI, (D) pristine DOX, (E) pristine MTX, (F) pristine MIT, (G) pristine 5FU, (H) pristine CIS, (I) Cyclo-HH, Zn^2+^, NO_3_^–^, and EPI, (J) Cyclo-HH, Zn^2+^, NO_3_^–^, and DOX, (K) Cyclo-HH, Zn^2+^, NO_3_^–^, and MTX, (L) Cyclo-HH, Zn^2+^, NO_3_^–^, and MIT, (M) Cyclo-HH, Zn^2+^, NO_3_^–^, and 5FU, and (N) Cyclo-HH, Zn^2+^, NO_3_^–^, and CIS at different excitation wavelength (280–560, 20 nm excitation wavelength step).

Fluorescence spectroscopy was employed to study the co-assembly of Cyclo-HH, Zn^2+^, and NO_3_^–^ with EPI, DOX, MTX, MIT, 5FU, or CIS, and the degree of drug encapsulation. For this purpose, fluorescence spectroscopy was employed also for each drug alone, the peptide alone, and the peptide with Zn^2+^ and NO_3_^-^. The fluorescence properties of the aforementioned individual and co-assembled molecules were measured using different excitation wavelengths from 280 to 560 at 20 nm intervals, as shown in (Figure 5). The appearance of a fluorescence peak at 308 nm at the three lowest excitation wavelengths, as well as the change in the spectra overall for excitation wavelengths larger than 360 nm upon adding Zn(NO_3_)_2_ to the Cyclo-HH dipeptide, indicated the importance of Zn^2+^ and NO_3_^–^ and their interactions with Cyclo-HH (Figure 5A,B). A comparison of spectra at excitation wavelengths larger than 360 nm provided additional insights into the co-assembling properties of drugs as well as Zn^2+^ and NO_3_^–^ with Cyclo-HH. Particularly, pristine EPI, DOX, and MTX are intrinsically fluorescent (Figure 5C–E), 5FU has lower intrinsic fluorescence (Figure 5G), while MIT and CIS have nearly no intrinsic fluorescence (Figure 5F,H). Interestingly, a comparison of the spectra between the pristine drugs and the nanostructures formed by the co-assembly of Cyclo-HH, Zn^2+^, NO_3_^–^ on one hand, versus the nanostructures formed by the co-assembly of Cyclo-HH, Zn^2+^, NO_3_^–^ and the six different drugs, on the other hand, suggests that EPI, DOX, and MTX, followed by MIT and 5FU (Figure 5I–M), and to a much lesser extent CIS (Figure 5N), have an impact on the nanostructures formed; thereby, this could serve as indication of enhanced co-assembly and encapsulation of EPI, DOX, and EPI, followed by MIT and 5FU, and to a much lesser extent CIS within the nanostructures (Figure 5I–N). The latter could presumably indicate that CIS is significantly less encapsulated and/or if it is encapsulated to a small extent, this could be at the exterior, which is in line with computational results. It is also important to note that the presence of drugs in the co-assembly process could influence the arrangement of Zn^2+^ and NO_3_^–^ in all cases, especially in the cases of enhanced encapsulation; this is evident from the high-fluorescence of Cyclo-HH, Zn^2+^, NO_3_^–^ and MTX (Figure 5K), which according to computations it is attributed to the strong interaction between MTX and Zn^2+^. Additionally, for EPI and DOX (Figure 5I,J), which are intrinsically fluorescent, the relative difference in fluorescence between their pristine form in comparison to their form within the co-assembled nanostructures could also be presumably attributed to the fact that at least a portion of the drugs is well-buried within the co-assembled nanostructures; this postulation is in line with the computational results.

### Drug Stability and Release from the Nanocarriers

**Figure 6:**
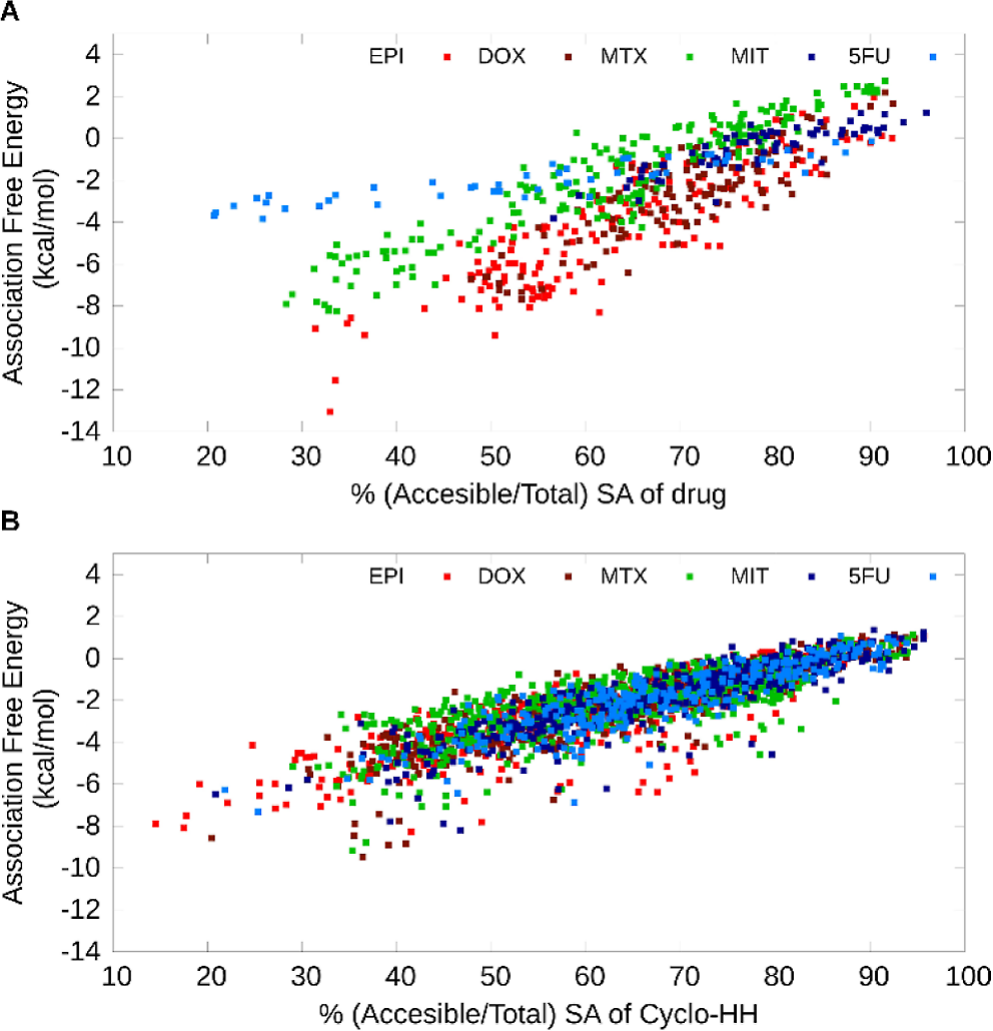
Association-free energy (kcal/mol) of (A) drugs and (B) Cyclo-HH peptides with a preformed co-assembled cluster as a function of the percentage of solvent accessible surface area out of the total surface area in the 20 highest complexity clusters of EPI (red), DOX (maroon), MTX (green), MIT (dark blue), and 5FU (light blue).

To assess the degree of stability of drugs bound within the formed clusters in the simulations and also in relation to their relative solvent exposure, we computationally studied the relationship between the association-free energy of a drug with the rest of the cluster as a function of its ratio of solvent accessible over the total accessible surface area. This analysis was performed for the 20 highest complexity clusters of each system and showed a nearly linear relationship between the two metrics for all drugs. This depicted that the more buried a drug was, the more favorable its binding was. Nevertheless, the linear relationships differ for clusters with EPI, DOX, and MTX versus MIT and 5FU, depicting that EPI, DOX, and MTX are more favorable to be assembled with the cluster compared to the drugs of the second group (Figure 6A) for well-buried drugs. Similarly, the association-free energy of the Cyclo-HH peptide with the rest of the system as a function of its ratio of solvent accessible over the total accessible surface area was also linear for all the simulated systems, which also depicts that the more buried the peptides were, the more favorable their binding was (Figure 6B). However, in this case, the same linear relationship holds for all systems. Overall, both graphs (Figure 6A,B) further indicate that a successfully co-assembled nanocarrier is an outcome of low association-free energy of Cyclo-HH to the system, in conjunction with low association free energy of the drug to the system. The most successful nanocarriers, incorporating EPI, DOX, and MTX, could be formed as an outcome of the ability of well-buried drugs to form interactions with the cluster (which includes peptides, drugs, and ions) that can compensate for the ability of well-buried peptides to form interactions with the cluster when both drugs and peptides are compared against the same degree of exposure. Furthermore, in all systems, solvent-exposed drugs showed higher association-free energies, whereas primarily in systems incorporating EPI, DOX, and MTX, drugs with low exposure showed considerably lower association-free energies (Figure 6A). Such strongly bound drugs within the clusters can potentially contribute significantly to gradual and slower release behavior. Figure 2 also presents a representative overview of the above, with EPI, DOX, and MTX being distributed throughout the clusters, while MIT, 5FU, and to a larger extent CIS, are mostly located in the exterior of the clusters.

**Figure 7:**
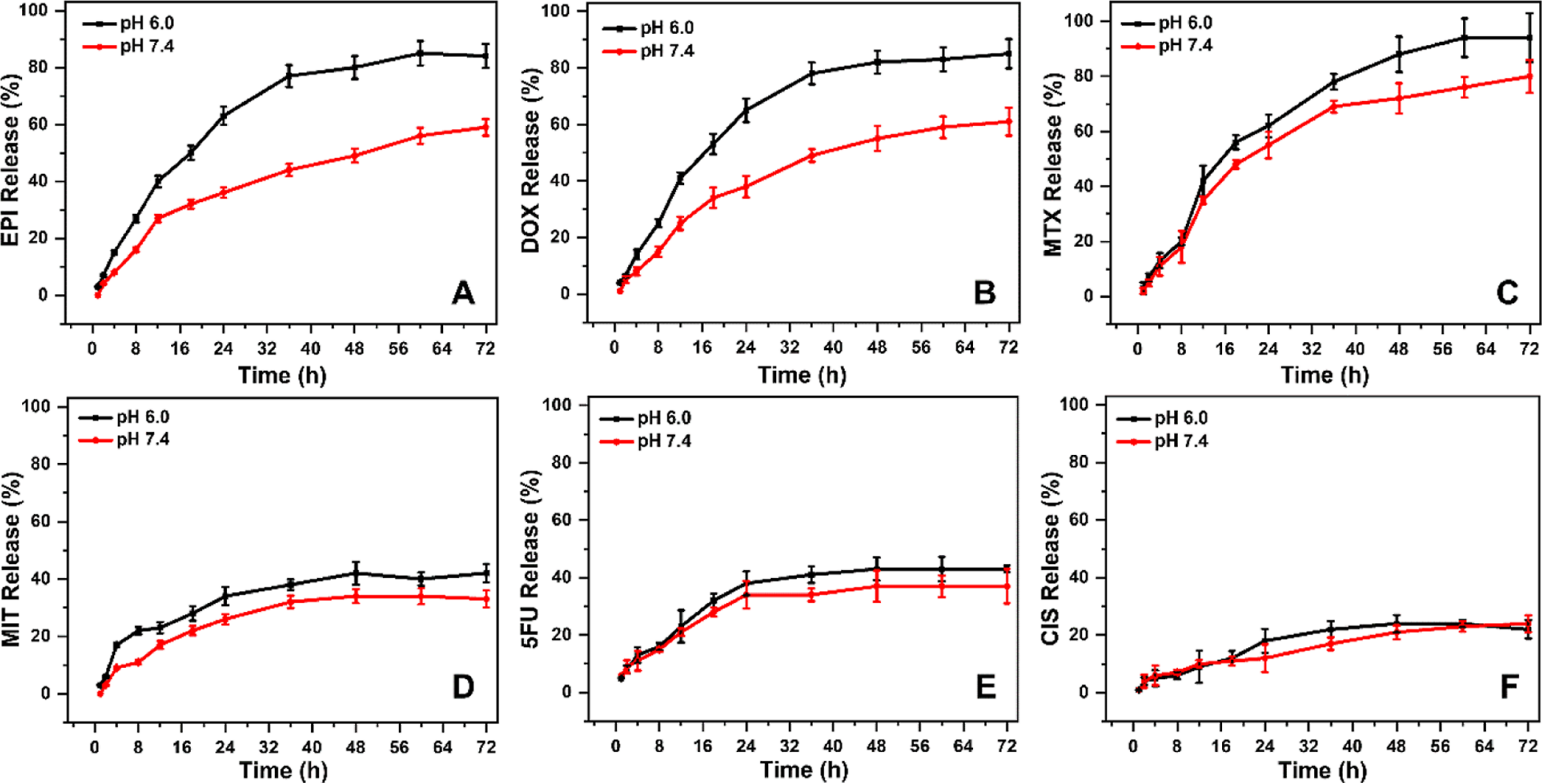
Drug release profiles of nanostructures formed by the co-assembly of Cyclo-HH, Zn^2+^, and NO_3_^–^ with (A) EPI, (B) DOX, (C) MTX, (D) MIT, (E) 5FU, and (F) CIS in 3.5 kDa dialysis chambers at two different pH values (pH 6.0 or 7.4).

We additionally experimentally studied the release profile of each drug encapsulated in the nanostructures of all six combinations of Cyclo-HH, Zn^2+^, and NO_3_^–^ with the drug by UV–vis absorption spectra at different time points (from 0 to 72 h). Drug release profile was observed for EPI, DOX, and MTX, with more efficient release observed in a slightly acidic environment (pH 6.0) compared to a slightly alkaline buffer (pH 7.4) (Figure 7A–C). Drug release was found to be faster in slightly acidic buffer solution compared to a neutral/alkaline buffer. The highest level of drug release was observed in the nanostructures formed by the co-assembly of Cyclo-HH, Zn^2+^, and NO_3_^–^ with MTX, followed by EPI and DOX. In these assays, the amount of drug initially added to the co-assembly mixture was defined as 100%. In the nanostructures formed by the co-assembly of Cyclo-HH, Zn^2+^, and NO_3_^–^ with MIT (Figure 7D), and 5FU (Figure 7E), approximately 40–50% of the drug was observed to be released after 72 h, while in the nanostructures formed by the co-assembly of Cyclo-HH, Zn^2+^ and NO_3_^–^ with CIS (Figure 7F) approximately less than 20% of the drug was observed to be released after 72 h. For MIT, 5FU, and CIS the amount released is similar at the two pH conditions, with pH 6.0 being overall higher than pH 7.4. Notably, the above results are in line with the fact that EPI, DOX, and MTX are better encapsulated compared by MIT and 5FU, followed by CIS at which the encapsulation could be minimal and primarily at the exterior, in line with the computational results (see above and below).

### Cytocompatibility Analysis and Nanocarrier Localization in Cultured Cells

We tested the cytotoxicity of the nanostructures formed by the co-assembly of Cyclo-HH, Zn^2+^, and NO_3_^–^ with EPI, DOX, MIT, MTX, 5FU, and CIS toward HeLa cells. Based on the results of the in vitro cell viability assay (Figure S18), the Cyclo-HH peptide, either alone or in the presence of Zn^2+^/NO_3_^–^, showed excellent cytocompatibility (Figure S18M,N), whereas each drug alone showed very toxic properties, as expected (Figure S18B, D, F, H, J, L). Furthermore, nanostructures of Cyclo-HH, Zn^2+^, and NO_3_^–^ with drugs formed by the co-assembly approach showed lower toxicity compared to the pristine drugs (Figure S18A-L).

**Figure 8:**
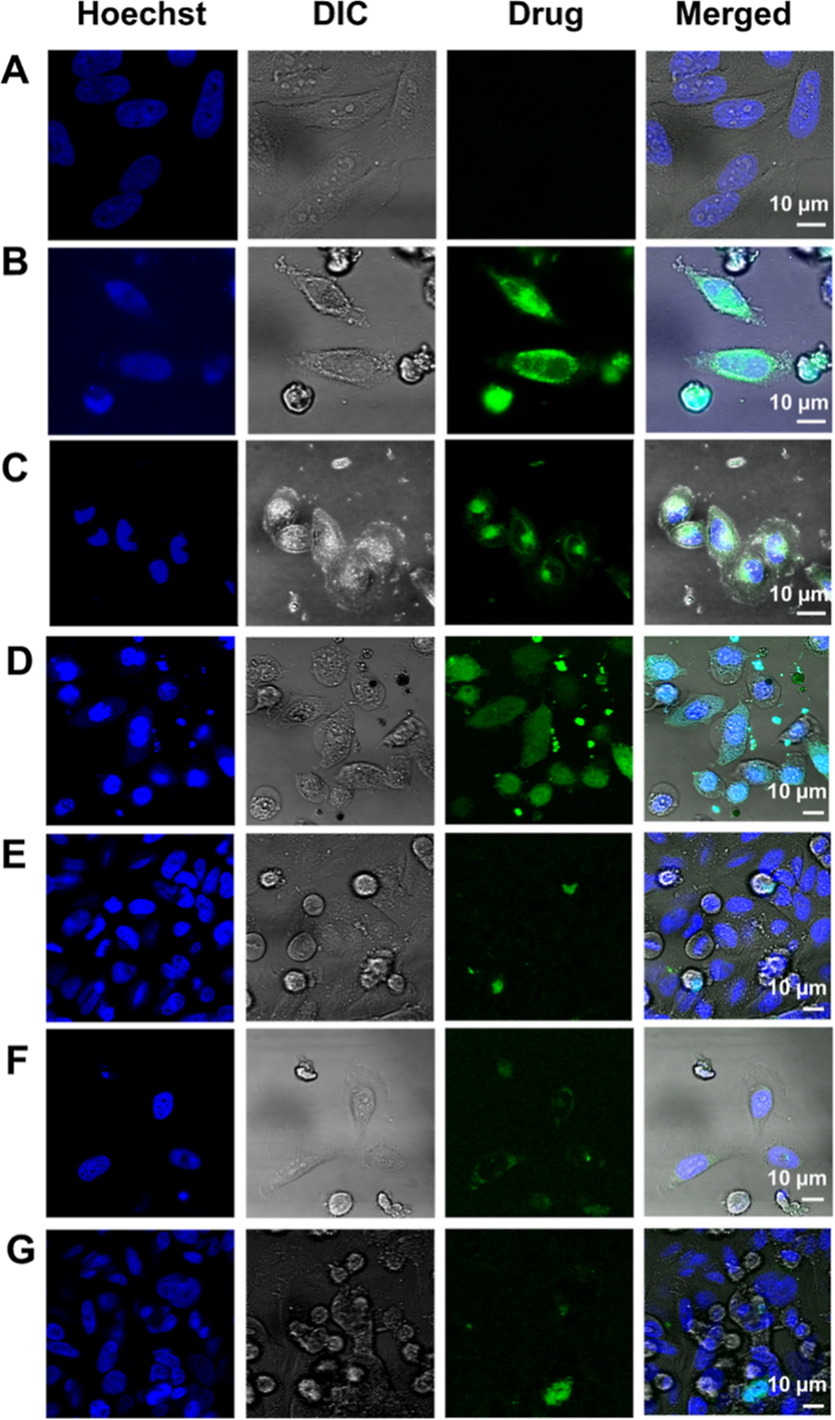
Live imaging of HeLa cells by confocal microscopy. (A) Control without any treatment. (B–G) After a 24 h incubation with Cyclo-HH, Zn^2+^, and NO_3_^–^ co-assembled with (B) EPI, (C) DOX, (D) MTX, (E) MIT, (F) 5FU, and (G) CIS.

Following the cytotoxicity assays, we examined the in vitro drug release via live imaging of Hela cells incubated with Cyclo-HH, Zn^2+^, and NO_3_^–^ with drug nanostructures formed by co-assembly for 24 h (Figure 8). The fluorescence intensities in cells treated with Cyclo-HH, Zn^2+^, and NO_3_^–^ with EPI (Figure 8B), Cyclo-HH, Zn^2+^, and NO_3_^–^ with DOX (Figure 8C) or Cyclo-HH, Zn^2+^, and NO_3_^–^ with MTX (Figure 8D) were significantly higher compared to cells treated with the corresponding pristine drugs after 24 h (Figure S19). These results suggest efficient uptake of EPI, DOX, and MTX by HeLa cells through the Cyclo-HH, Zn^2+^, and NO_3_^–^ nanostructures using the co-assembly approach, indicating their potential use as imaging or therapeutic tools. Comparing the results of the Cyclo-HH, Zn^2+^, and NO_3_^–^ with MIT (Figure 8E), and Cyclo-HH, Zn^2+^, and NO_3_^–^ with 5FU (Figure 8F) nanostructures formed by co-assembly, the amount of drug entering the cell appears to be low, which can be a combination of low fluorescence from the drugs and the nanocarriers, with lower extent of encapsulation compared to MTX, EPI and DOX. Furthermore, very low fluorescence was observed in cells treated with Cyclo-HH, Zn^2+^, and NO_3_^–^ with CIS (Figure 8G) nanostructures formed by co-assembly which also might be due to a combination of low fluorescence from the drug and the nanocarriers, in combination with the significantly lower extent of encapsulation compared to all other drugs. Moreover, based on computational and experimental results, MTX, EPI, and DOX showed higher encapsulation efficiency in comparison to others. In line with this, in vitro cellular studies demonstrated colocalization with the HeLa cells.

## Discussion

Cancer is a serious health problem and is a complex disease. Drug delivery to cancer cells is one of the key needs for cancer therapy.^55^ Nanobased drug delivery is advantageous compared to conventional drugs.^56^ Nanobased drugs can potentially be more stable and biocompatible, possess enhanced permeability and retention effect, as well as combine precise targeting.^56^ Toxicology-related issues are important to be addressed as part of new improved cancer therapeutic strategies in addition to combination therapy for different types of cancer which also needs critical consideration.^15^ Considering the diversity of mechanisms related to cancer, combination therapy with nanomaterial-based drug carriers is a subject of much needed crucial future investigation at preclinical and clinical level.^15^

Self-assembled peptide materials possess a series of advantageous properties, particularly in their ability to form different types of nanostructures which can potentially serve as drug nanocarriers for drug release applications.^1,2^ Therefore, a promising direction in cancer therapeutics is the design of new peptide materials that can be self-assembled for the encapsulation of cancer drugs.^1^ The intrinsic benefits of peptide self-assembled materials and the growing advances in computational and experimental approaches in the study and design of self-assembled systems^57−66^ could serve as a means to design new classes of cancer drug delivery systems which may provide further alternatives to existing approaches. Additionally, they can serve as stepping stones for designing systems comprising peptide self-assembled materials, along with other materials for the delivery of cancer drugs.

Prompted by our recent studies showing Cyclo-HH co-assembling with cancer drug EPI in the presence, of Zn^2+^ and NO_3^-^_,^22^ in this study we aimed to systematically investigate the potential encapsulation properties, of different drugs with diverse physicochemical properties, by the same system, i.e., Cyclo-HH, Zn^2+^, and NO_3_^–^. Thus, we investigated the co-assembly properties of Cyclo-HH, Zn^2+^, and NO_3_^–^ with six cancer drugs, EPI, DOX, MTX, MIT, 5FU, and CIS using a combination of computational and experimental methods. Computations focused on the use of simulations, followed by in-depth structural and energetic analysis, while experiments focused on the use of TEM, fluorescence, and confocal microscopy to provide insights into drug encapsulation, drug release, and cell viability. Computations aimed to study and compare, atomistically, the early stages of nanocarrier formation and properties for different cancer drugs. Therefore, investigating simulation clusters with a sufficiently large number of entities was considered beneficial as these could represent co-assembled clusters of higher complexity, which could be more likely related to the assemblies observed experimentally. The computational setup used in conjunction with the ability to reach relatively long simulation duration times was key in achieving this, in accordance with experiments. In tandem, computational and experimental studies depict that EPI, DOX and MTX, and to a lesser extent MIT, and 5FU, have the capacity to co-assemble with Cyclo-HH, Zn^2+^, and NO_3_^–^ ions, while a significantly lower propensity was observed for CIS. EPI, DOX, and MTX have improved drug encapsulation and drug release properties, followed by MIT and 5FU.

The highest level of drug release was observed for Cyclo-HH, Zn^2+^, and NO_3_^–^ co-assembled with EPI, DOX, and MTX, potentially as a result of higher drug encapsulation. In the case of MIT and 5FU, approximately 40–50% of the drug was observed to be released after 72 h. In contrast, Cyclo-HH, Zn^2+^, and NO_3_^–^ with CIS showed the release of less than 20% of the drug. Furthermore, drug release was found to be faster in a slightly acidic buffer solution compared with a neutral/alkaline buffer. This can be considered an advantageous property of the co-assembled systems and could be potentially attributed to the fact that in slightly acidic conditions, the imidazole ring becomes partly protonated, resulting in easier decomposition of the co-assembled structures. The importance of the imidazole ring for its coordination with Zn^2+^ and NO_3_^–^ in the ability of Cyclo-HH to self-assemble was discussed in the past,^21−23^ and is examined and highlighted extensively in this study. Importantly in the current work, we underline the key combined role of ions Zn^2+^, NO_3_^–,^ as well as Cyclo-HH, in the co-assembly with drugs, in combination with their influence on fluorescence properties.

The coordination of NO_3_^–^ with EPI and DOX facilitates co-assembly, and impacts the role of Zn^2+^ and Cyclo-HH in the co-assembly; additionally, the coordination of Zn^2+^ with MTX facilitates co-assembly and augments fluorescence, and similarly impacts the role of NO_3_^–^ and Cyclo-HH. In all systems, with the exception of CIS, additional nonspecific interactions between Zn^2+^ and NO_3_^–^, Cyclo-HH and the drugs also occur and stabilize the co-assembled nanostructures. The particular aforementioned interactions between NO_3_^–^ with EPI and DOX, as well as between Zn^2+^ with MTX, seem to be the key factors for the improved co-assembled properties associated with these drugs. Our energetic analysis depicts that the higher the degree of drug burial, the lower the association of the drug is for EPI, DOX, MTX, MIT, and 5FU. Importantly though, the association-free energy for higher degree of drug burial becomes significantly lower for EPI, DOX, and MTX and comparable to the association-free energy of Cyclo-HH, compared at the same degree of burial. Thus, the advantageous co-assembling properties of EPI, DOX, and MTX could be attributed to the ability of well-buried drugs to “fairly compete” with well-buried peptides in their interactions with the rest of the system. This is also in line with our additional structural analysis depicting that the tendency of drug–drug and drug-peptide interactions, directly and/or indirectly enabled by Zn^2+^ and NO_3_^–^, is amplified in systems with EPI, DOX, and MTX. In general, the favorable association of free energies in combination with higher degree of drug burial, also correlate with the fact that at least a portion of EPI, DOX, and MTX drugs in the co-assembled nanostructures could have partly lower release rates.

## Conclusions

Overall, in this study, we used computations and experiments to investigate the co-assembly of a particular system comprising Cyclo-HH, Zn^2+^, and NO_3_^–^ with different cancer drugs namely EPI, DOX, MTX, MIT, 5FU, and CIS. Our results demonstrated that EPI, DOX, and MTX can successfully co-assemble with Cyclo-HH, Zn^2+^, and NO_3_^–^. The MD simulations, supported by experimental observations, uncovered the primary molecular interactions that contributed to the formation of co-assembled nanostructures. In summary, our understanding on the key properties leading to enhanced co-assembly for particular cases is crucial and can enable future studies in the use of computational approaches in the *de novo* design of novel systems combining efficient co-assembly with different cancer drugs, in addition to additional critical properties required for cancer drug delivery systems.^1^

## Supporting Information Available

Supporting Information includes four sections: Supporting Methods (additional details on the computational methods), Supporting Table S1 (results of the SVM model), Supporting Figures (Figure S1: decomposition of different chemical groups of Cyclo-HH and the drugs; Figures S2–S17, and Figures S18–19: additional computational and experimental results, respectively), and Supporting References (PDF)
Coordinates of high-complexity clusters in PDB format (ZIP)

## Notes

PDB coordinates of examples of high-complexity clusters formed per simulated system, except for CIS.
