# Supplementary material for: Co-Assembly of Cancer Drugs with Cyclo-HH Peptides: Insights from Simulations and Experiments
Source: ACS Appl Bio Mater. 2024 Mar 13;7(4):2309–24. doi: 10.1021/acsabm.3c01304 (PMC11022239; doi:10.1021/acsabm.3c01304)
Supplement: Supplementary file 1 — mt3c01304_si_001.pdf [file mt3c01304_si_001.pdf]

Co-assembly of Cancer Drugs with Cyclo-HH peptides:
Insights from Simulations and Experiments

Supporting Information

Anastasia Vlachou[1#], Vijay Bhooshan Kumar[2,3,4,#], Om Shanker Tiwari[2,3,4,#], Sigal Rencus-Lazar[2,3,4], Yu Chen[2,3,4], Busra Ozguney[1], Ehud Gazit[2,3,4]*, Phanourios Tamamis[1,5]*

[1]Artie McFerrin Department of Chemical Engineering, Texas A&M University, College Station, Texas 77843-3122, United States.

[2]The Shmunis School of Biomedicine and Cancer Research, George S. Wise Faculty of Life Sciences, Tel Aviv University, Tel Aviv 6997801, Israel.

[3]Department of Materials Science and Engineering, Iby and Aladar Fleischman Faculty of Engineering, Tel Aviv University, Tel Aviv 6997801, Israel.

[4]Sagol School of Neuroscience, Tel Aviv University, Tel Aviv 6997801, Israel.

[5]Department of Materials Science and Engineering, Texas A&M University, College Station, Texas 77843-3003, United States.

[#] Equally contributing first authors

* Corresponding authors:
Ehud Gazit: ehudga@tauex.tau.ac.il
Phanourios Tamamis: tamamis@tamu.edu

Table of Contents

- Supporting Methods
- Supporting Tables
- Supporting Figures
- Supporting References

Supporting Methods

Supporting Information on Structural and Energy Analysis of the Simulated Systems: The following section provides a detailed description of the calculations performed to obtain data presented in particular figures and tables. As mentioned in the main text, the analysis below is valid for clusters with 30 or more peptides, ions and drugs, detected with snapshots extracted every 1 ns. All analyses were performed using in-house FORTRAN codes, in conjunction with other programs Wordom[1,2], CHARMM[3], and Autodock4Zn[4].

The **percentage probability of drug encapsulation** was calculated as the fraction of clusters with at least one drug divided by the number of all clusters formed per system. Additionally, the percentage probability of drug encapsulation was calculated as the fraction of clusters with at least two drugs divided by the number of all clusters formed per system. Also, we calculated the percentage probability of no drug encapsulation as the fraction of clusters with no drugs divided by the number of all clusters formed per system. The results are presented in **Figure 1B**. In what follows the analysis focuses on clusters containing at least one drug; in particular cases, analysis was performed also for clusters containing no drug, for comparison purposes. The data in the graphs present the average value and population standard deviation as a function of clusters' size (which was defined in the main text), and the clusters were divided based on the size into bins of 30-39, 40-49, 50-59, 60-69, 70-79, 80-89, 90-99, 100-109, 110-119, 120-129, 130-139 and 140-149. It is worth noting that all the calculations related to interacting peptides, drugs and ions were performed under the following condition: if a peptide, drug or ion interacts with two or more peptides, drugs or ions, all interactions are considered individually, which means that all interactions were considered, given that there is at least an atom pair between the two molecules within a 3.5 Å cutoff.

The **percentage of drug encapsulation** for each cluster was calculated as the fraction of the number of drugs encapsulated in the cluster divided by the total number of drugs available in the simulated system, and the data are presented in **Figure 1C**.

In what follows, we provide a detailed description of the structural properties of the formed clusters extracted and analyzed as a function of clusters' size:

(i) The **percentage composition** per component (Cyclo-HH, drug, Zn^{2+}, and NO_3^-) for each given cluster was calculated as the fraction of the number of peptides, drugs and ions in the cluster respectively, divided by the sum of the total number of peptides, drugs and ions comprising the cluster, and the results are presented in **Figure S2**. Additional calculations were performed for the clusters without any drugs (containing only Cyclo-HH peptides, Zn^{2+}, and NO_3^-) and the results are presented in **Figure S2** for comparison purposes.

(ii) The **probability of each Cyclo-HH peptide interacting with other peptides, drugs and ions** was calculated as follows. For each Cyclo-HH peptide in each given cluster, we calculated the number of interactions with: (a) other Cyclo-HH peptides, (b) drugs, (c) Zn^{2+}, and (d) NO_3^- , divided by the total number of Cyclo-HH peptides in the cluster, and the results are presented in **Figure S3**. Additional calculations were performed for the clusters without any drugs (containing only Cyclo-HH peptides, Zn^{2+}, and NO_3^-) and the results are presented in **Figure S3** for comparison purposes. The **probability of each drug interacting with other drugs, peptides and ions** was calculated as follows. For each drug in each given cluster, we calculated the number of interactions with: (a) other drugs, (b) Cyclo-HH peptides, (c) Zn^{2+}, and (d) NO_3^- , divided by the total number of drugs in the cluster, and the results are presented in **Figure S8**. It is important to remember that within the calculations mentioned above, if a peptide or, drug or ion interacts with two or more other peptides, drugs and ions, all interactions are considered individually, which means that all interactions were considered, given that there is at least an atom pair between the two peptides, drugs or ions within a 3.5 Å cutoff. This explains why some values of **Figure S3 and Figure S8** are larger than 1.

(iii) The **percentage ratio of solvent accessible surface area divided by the total surface area** per component was calculated as follows. For each Cyclo-HH peptide, drug, NO_3^-, and Zn^{2+} in each given cluster, we calculated the fraction of the solvent accessible surface area (SASA) of this as part of the cluster ($Å^2$) divided by the total surface area (TSA) ($Å^2$) of the same in the absence of the rest cluster's peptides, drugs and ions. Following, for each component, we calculated the sum of the fractions over all peptides, drugs and ions (respectively) and normalized it by the number of all peptides, drugs and ions (respectively) in the cluster; and the results are presented in **Figure S4**. Additional calculations were performed for the clusters without any drugs (containing only Cyclo-HH peptides, Zn^{2+}, and NO_3^-) and the results are presented in **Figure S4** for comparison purposes. For all the solvent-accessible surface area calculations, we used Wordom[5,1,2] ; and the probe radius of IPA 2.5 Å[6,7]. It is worth

noting that the clusters' structures given in Wordom [1,2] were collected and stored using an in-house FORTRAN code.

(iv) The **radius of gyration (Å)** for each given cluster was calculated using the Wordom [1,2] tool. For the calculations, the atoms of all peptides, drugs and ions in the cluster were considered and the results are presented in **Figure S5**. Additional calculations were performed for the clusters without any drugs (containing only Cyclo-HH peptides, Zn^{2+}, and NO_3^-) and the results are presented in **Figure S5** for comparison purposes. It is worth noting that the clusters' structures given in Wordom 1,2 were collected and stored using an in-house FORTRAN code.

The **ratio of the drugs divided by the Cyclo-HH peptides** for each given cluster was calculated as the fraction of the number of the drugs in the cluster divided by the number of Cyclo-HH peptides in the cluster, and the results are presented in **Figure S6**.

The **ratio of Zn^{2+} divided by the Cyclo-HH peptides** for each given cluster was calculated as the fraction of the number of the Zn^{2+} in the cluster divided by the number of Cyclo-HH peptides in the cluster, and the results are presented in **Figure S7**. Additional calculations were performed for the clusters without any drugs (containing only Cyclo-HH peptides, Zn^{2+}, and NO_3^-) and the results are presented in **Figure S7** for comparison purposes.

The **probability of a drug to mediate interactions with Cyclo-HH peptides or drugs** for each given cluster, referred to as XDY (where D: is a drug, X and Y: can be a drug or Cyclo-HH peptide), was calculated as the fraction of the number of the interactions at which the middle is a drug (D) mediating two other molecules, which can be another drug (D) or a Cyclo-HH peptide (P); divided by the total number of drugs in the cluster. All the possible mediated interactions by the drug are the following: PDP, PDD, and DDD. The results are presented in **Figure S9**.

The **probability of a Cyclo-HH peptide to mediate interactions with Cyclo-HH peptides or drugs** for each given cluster, referred to as XPY (where P: is a Cyclo-HH peptide, X and Y: can be drug or Cyclo-HH peptide), was calculated as the fraction of the number of the interactions at which the middle is a Cyclo-HH peptide (P) mediating two other molecules, which can be another Cyclo-HH peptide (P) or a drug (D); divided by the total number of

Cyclo-HH peptides in the cluster. All the possible mediated interactions by Cyclo-HH are the following: PPP, PPD, and DPD. The results are presented in **Figure S10**.

The **probability of a Zn^{2+} to mediate interactions with Cyclo-HH peptides or drugs** for each given cluster, referred to as XZY (where Z: is a Zn^{2+} , X and Y: can be drug or Cyclo-HH peptide), was calculated as the fraction of the number of the interactions at which the middle is a Zn^{2+} (Z) mediating two other molecules, which can be a drug (D) or a Cyclo-HH peptide (P); divided by the total number of Zn^{2+} in the cluster. All the possible mediated interactions by Zn^{2+} are the following: PZP, PZD, and DZD. The results are presented in **Figure S11**.

The **probability of a NO_3^- to mediate interactions with Cyclo-HH peptides or drugs** for each given cluster, referred to as XNY (where N: is a NO_3^-, X and Y: can be drug or Cyclo-HH peptide), was calculated as the fraction of the number of the interactions at which the middle is a NO_3^- (N) mediating two other molecules, which can be a drug (D) or a Cyclo-HH peptide (P); divided by the total number of NO_3^- in the cluster. All the possible mediated interactions by NO_3^- are the following: PNP, PND, and DND. The results are presented in **Figure S12**.

The **SVM model** which was applied is a multiclass Support Vector Machine (SVM) model using binary Kernel learners in Matlab and it was fed by the following features of each formed cluster with size larger than 30 co-assembled Cyclo-HH, drugs, NO_3^- , and Zn^{2+} and with at least one drug encapsulated:

**Features**
[PPP] mediated interactions
[PPD] mediated interactions
[PDP] mediated interactions
[PZP] mediated interactions
[PNP] mediated interactions
[PDD] mediated interactions
[PZD] mediated interactions
[PND] mediated interactions
[DDD] mediated interactions
[DPD] mediated interactions
[DZD] mediated interactions
[DND] mediated interactions
(Solvent Accessible Surface Area) / (Total Surface Area) of Cyclo-HH
(Solvent Accessible Surface Area) / (Total Surface Area) of drug
(Solvent Accessible Surface Area) / (Total Surface Area) of Zn^{2+}
(Solvent Accessible Surface Area) / (Total Surface Area) of NO_3^-

The classes were defined as follows: (i) 1st class = clusters of Epirubicin and Doxorubicin, (ii) 2nd class = clusters of Methotrexate, (iii) 3rd class = clusters of Mitomycin-D and 5-Fluorouracil. Out of all data, 75% were used for training and the rest for cross-validation. The chosen design code was the one-vs-one, according to which the multiclass SVM model is split into K(K – 1)/2 binary SVM models, where K represents the number of different unique classes. The results are presented in **Figure S13.**

The **probability of a Cyclo-HH chemical group to interact with Zn^{2+}** for each Cyclo-HH chemical group in each given cluster was calculated as the fraction of the number of the Cyclo-HH chemical groups (Figure S1) interacting with Zn^{2+} divided by the number of the Cyclo-HH-Zn^{2+} interactions occurring in the cluster; and the results are presented in **Figure S14.**

The **probability of a Cyclo-HH chemical group to interact with NO_3^-** for each Cyclo-HH chemical group in each given cluster was calculated as the fraction of the number of the Cyclo-HH chemical groups (Figure S1) interacting with NO_3^- divided by the number of the Cyclo-HH-NO_3^- interactions occurring in the cluster; and the results are presented in **Figure S15**.

The **probability of a drug chemical group to interact with Zn^{2+}** for each drug's chemical group in each given cluster was calculated as the fraction of the number of the drug's chemical groups (Figure S1) interacting with Zn^{2+} divided by the number of the drug - Zn^{2+} interactions occurring in the cluster; and the results are presented in **Figure S16**.

The **probability of a drug chemical group to interact with NO_3^-** for each drug's chemical group in each given cluster was calculated as the fraction of the number of the drug's chemical group (Figure S1) interacting with NO_3^- divided by the number of the drug - NO_3^- interactions occurring in the cluster; and the results are presented in **Figure S17**.

The **association free energy (kcal/mol)** of a drug with a preformed co-assembled cluster composed of the rest of peptides, drugs and ions, was calculated for each drug of the twenty highest complexity clusters using Autodock4Zn[4], after minimizing each cluster on CHARMM[3]. The energy minimization of each cluster was performed in a vacuum using 200 steps of the Steepest Descent (SD) algorithm, followed by 200 steps of the Adopted Basis Newton-Raphson (ABNR) algorithm, and ended by 200 steps of the Steepest Descent (SD) algorithm. The results are presented in **Figure 6A.** Additional calculations were performed for each Cyclo-HH peptide of the twenty highest complexity clusters and the results are presented in **Figure 6B.**

Supporting Tables

Table S1. The absolute values of the beta factors of each feature, according to the SVM model, sorted in descending order, are presented below for each binary system, along with the SVM model's accuracy, sensitivity and specificity.

**1st Class vs 2nd Class**		**1st Class vs 3rd Class**		**2nd Class vs 3rd Class**	
Feature	| Beta Factor |	Feature	| Beta Factor |	Feature	| Beta Factor |
(SASA/TSA) of Zn^{2+}	14.2844	(SASA/TSA) of NO_3^-	12.8767	DZD	9.4903
(SASA/TSA) of NO_3^-	12.7639	(SASA/TSA) of Zn^{2+}	12.4372	(SASA/TSA) of NO_3^-	6.9558
DZD	10.7703	(SASA/TSA) of drug	6.4077	PZD	6.5434
PZD	7.0361	DND	5.9678	(SASA/TSA) of Zn^{2+}	6.0867
PZP	6.5333	PNP	4.6461	DND	5.3269
PNP	2.7817	PZP	4.4997	(SASA/TSA) of drug	4.0171
(SASA/TSA) of drug	2.7507	PND	4.2073	DDD	1.5929
PND	2.0828	(SASA/TSA) of Cyclo-HH	1.9594	PZP	1.2354
(SASA/TSA) of Cyclo-HH	2.0555	DDD	0.7778	PNP	0.7576
DDD	0.3311	PPP	0.4392	PDD	0.5704
DPD	0.2669	PDD	0.4331	PDP	0.4052
DND	0.2158	PPD	0.1842	PPD	0.387
PDD	0.1674	PDP	0.183	DPD	0.2529
PDP	0.1527	DPD	0.1227	PND	0.2528
PPP	0.0778	PZD	0.0505	PPP	0.1946
PPD	0.0608	DZD	0	(SASA/TSA) of Cyclo-HH	0.1363
**Accuracy**				89%	
**Sensitivity**				96%	
**Specificity**				87%	

Supporting Figures

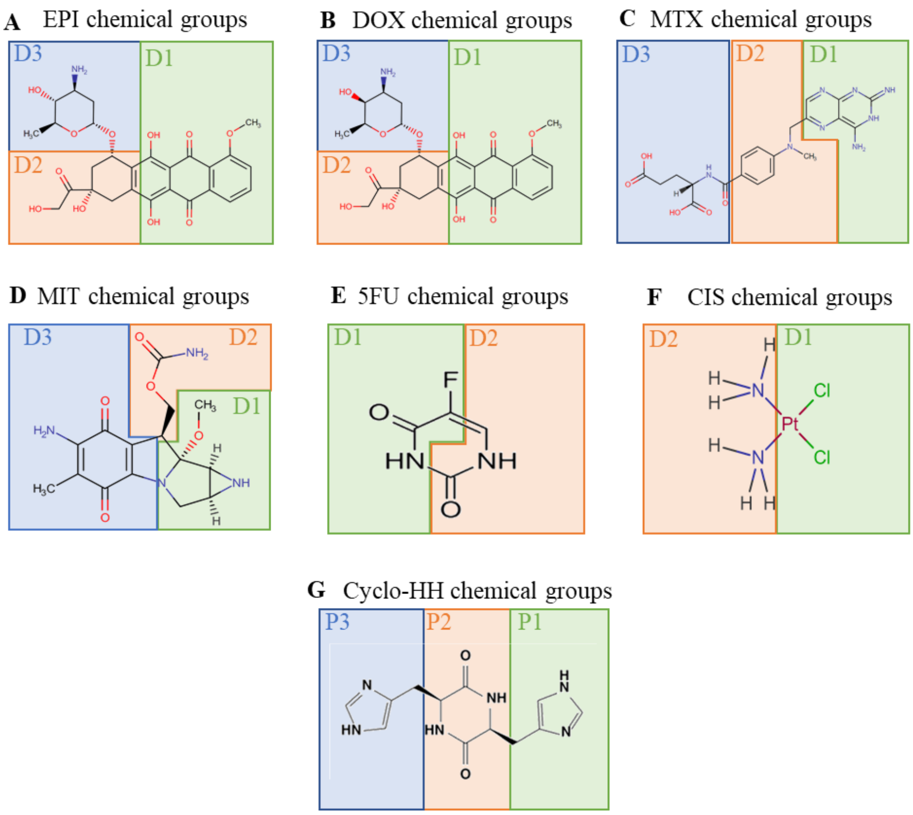

Figure S1. The drugs (A-F) and Cyclo-HH (G) were defined into chemical groups (D1-D3) and (P1-P3), as shown above, in different colors. The decomposition enabled our analysis and understanding of particular interactions between chemical groups of the drugs and Cyclo-HH, with each other, and with ions.

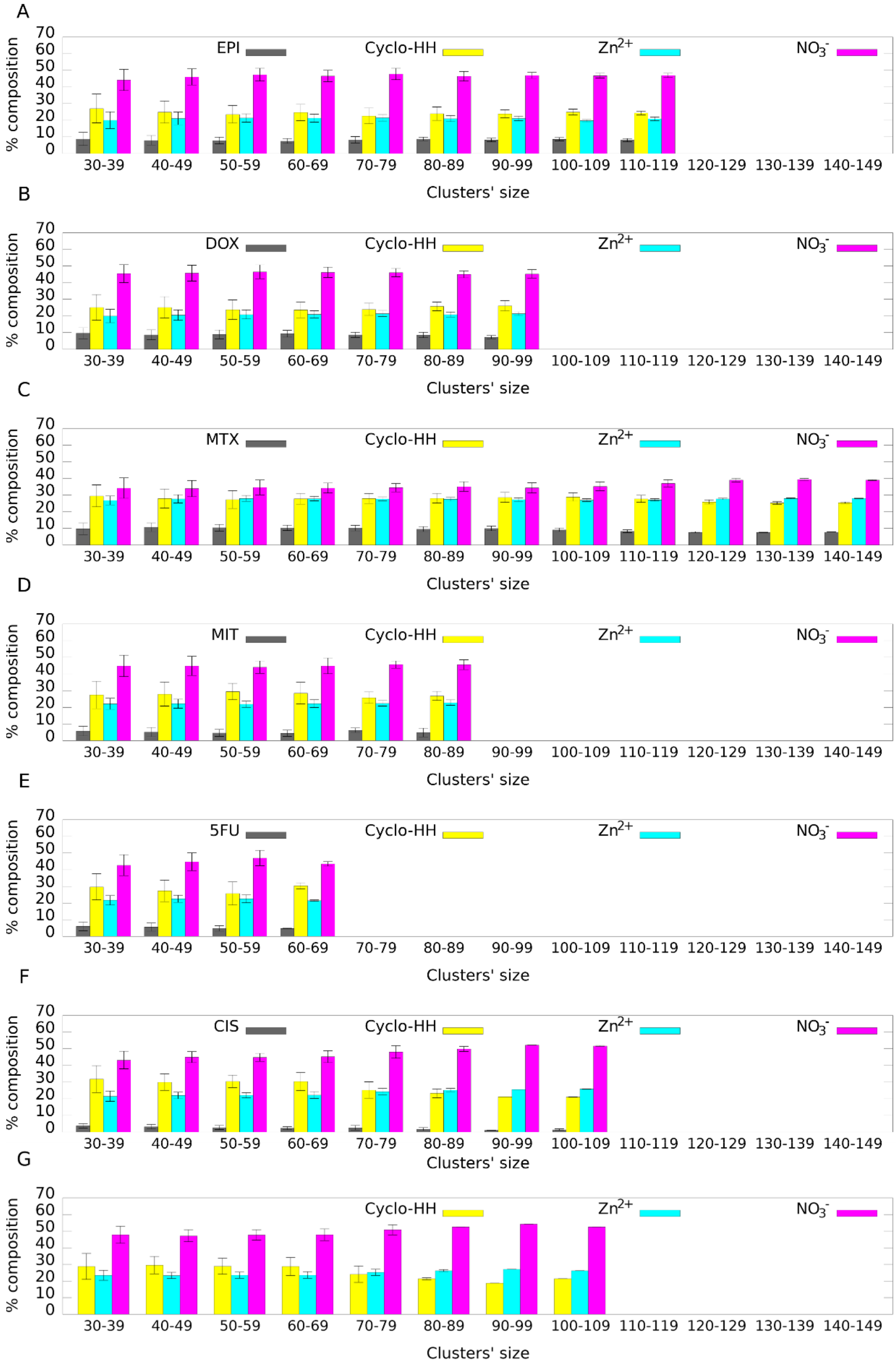

Figure S2. The percentage clusters' composition of all components: drugs (gray), cyclo-HH (yellow), Zn^{2+} (cyan) and NO_3^- (violet) as a function of the clusters' size for clusters with: (A) EPI, (B) DOX, (C) MTX, (D) MIT, (E) 5FU, (F) CIS and (G) no drugs.

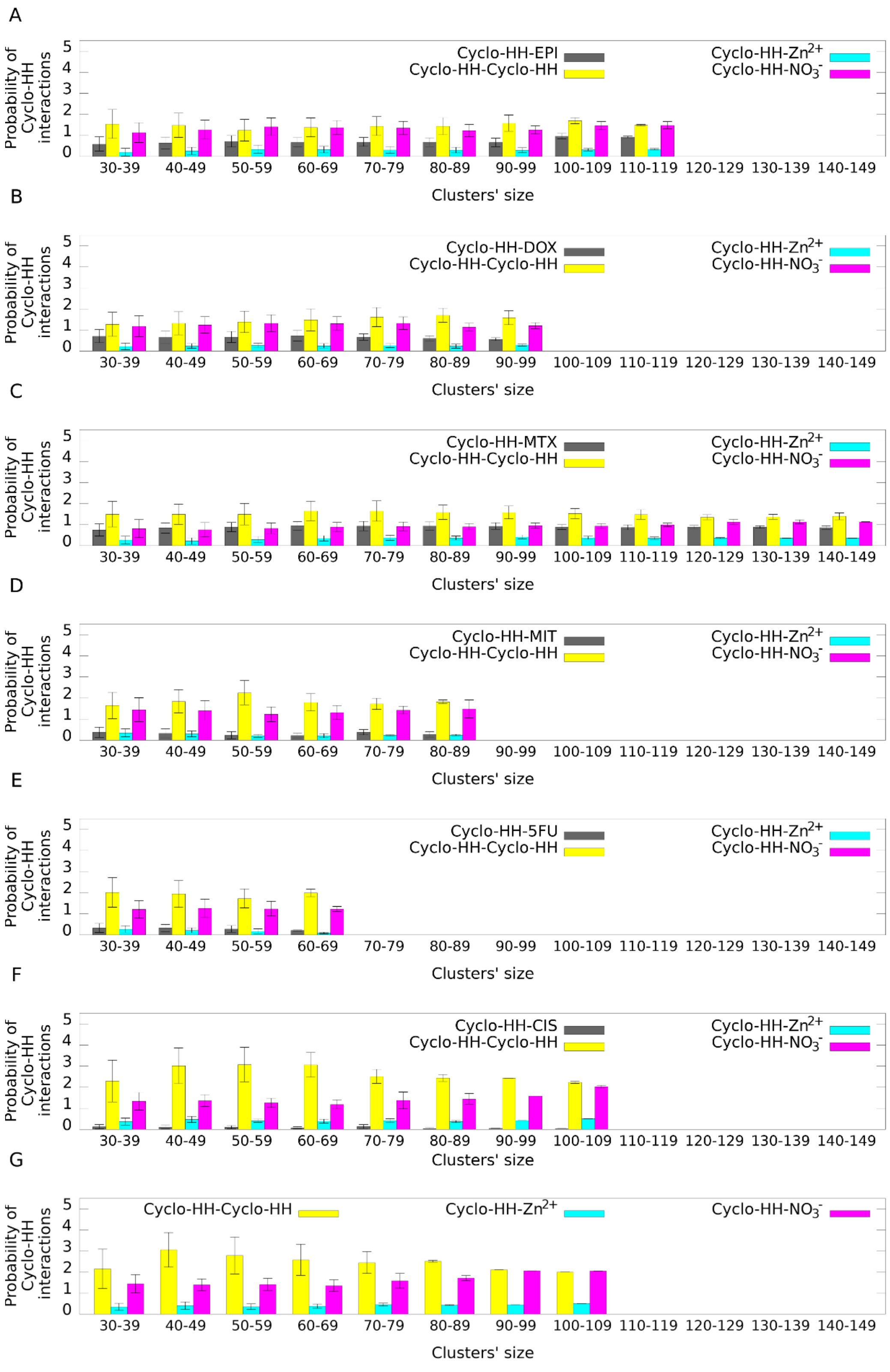

Figure S3. The probability of a Cyclo-HH peptide to interact with: drugs (gray), other Cyclo-HH peptides (yellow), Zn^{2+} (cyan) and NO_3^- (violet), as a function of the clusters' size for clusters with: (A) EPI, (B) DOX, (C) MTX, (D) MIT, (E) 5FU, (F) CIS and (G) no drugs.

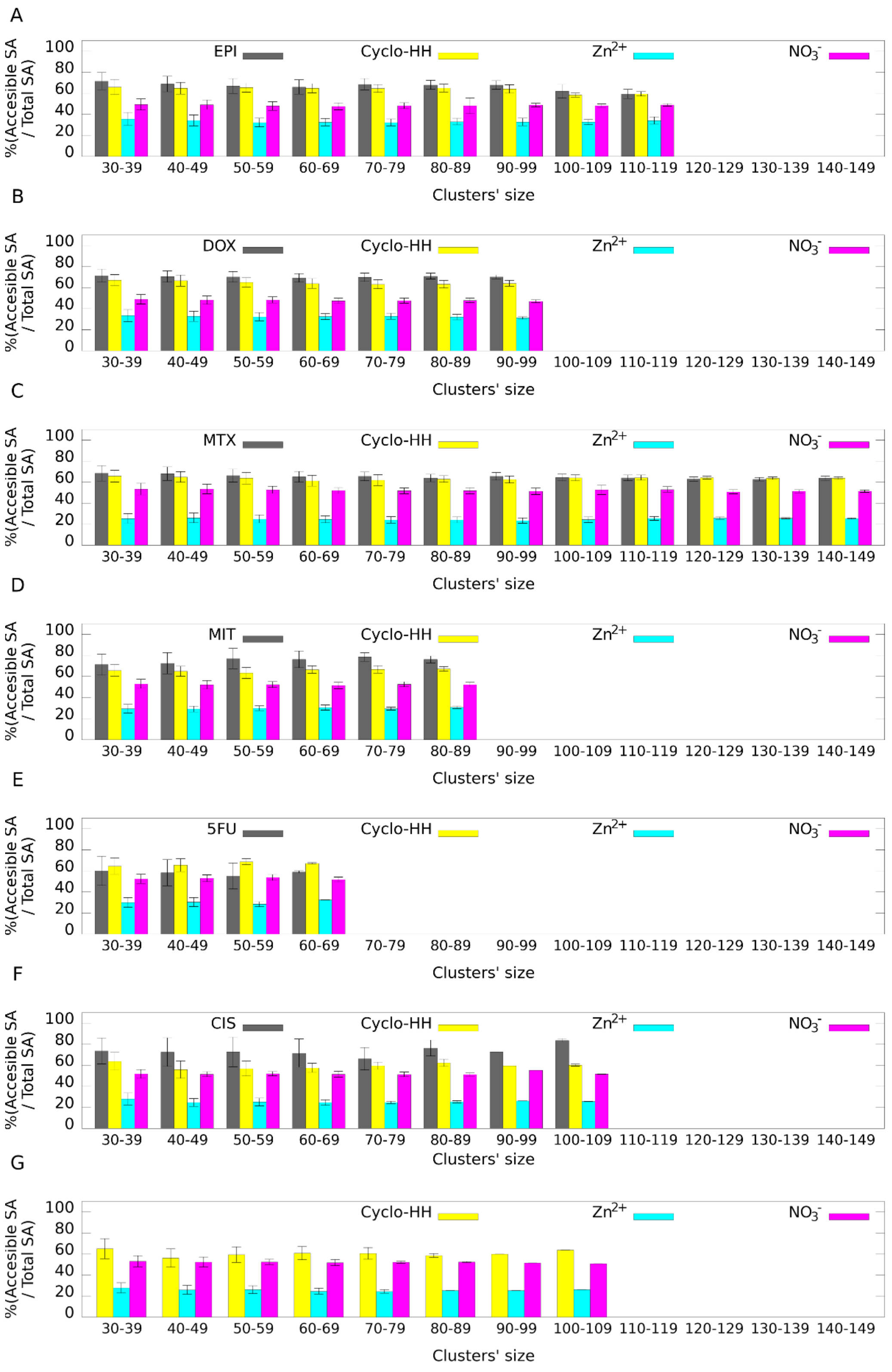

Figure S4. The percentage ratio of the solvent accessible surface area divided by the total accessible surface area per component: drug (gray), cyclo-HH (yellow), Zn^{2+} (cyan) and NO_3^- (violet) as a function of the clusters' size for clusters with (A) EPI, (B) DOX, (C) MTX, (D) MIT, (E) 5FU, (F) CIS and (G) no drugs

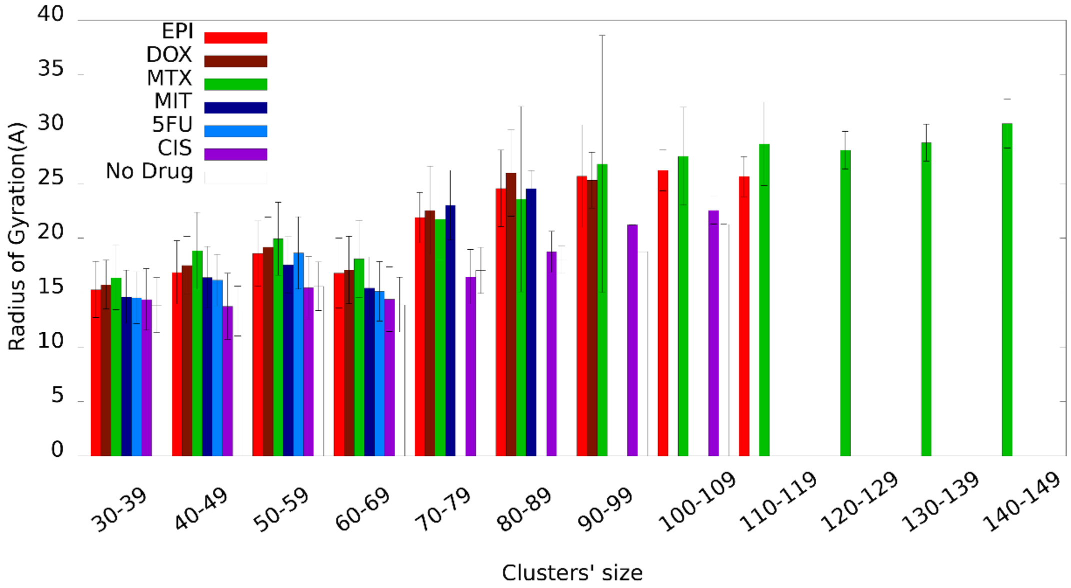

Figure S5. The average radius of gyration (Å) of clusters as a function of clusters' size for clusters with: EPI (red), DOX (maroon), MTX (green), MIT (dark blue), 5FU (light blue), CIS (purple) and no drugs (colorless).

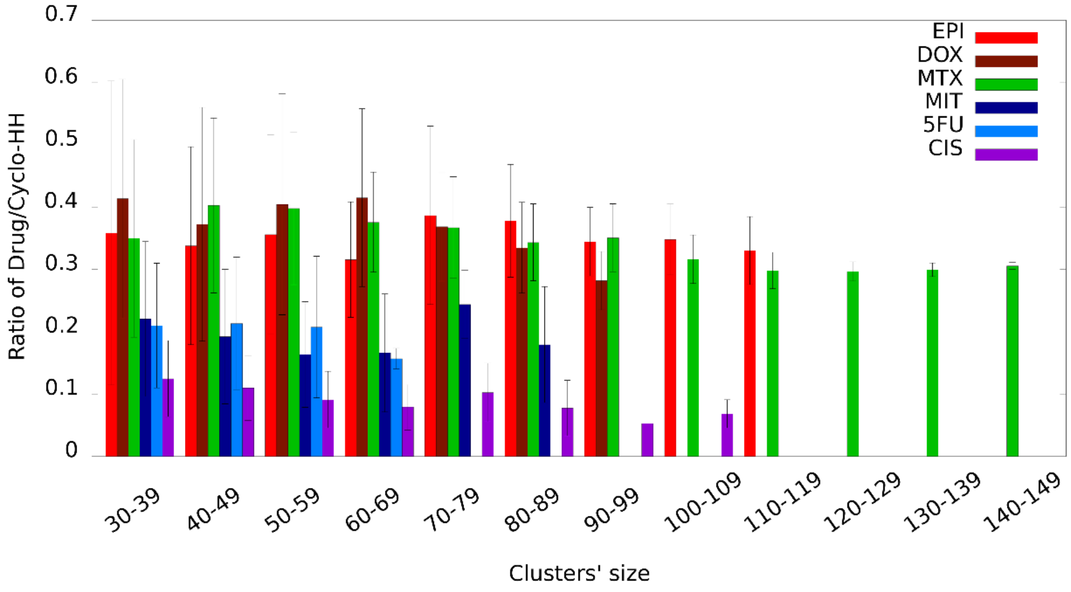

Figure S6. The ratio of drugs divided by Cyclo-HH peptides in the clusters as a function of clusters' size for clusters with: EPI (red), DOX (maroon), MTX (green), MIT (dark blue), 5FU (light blue), CIS (purple).

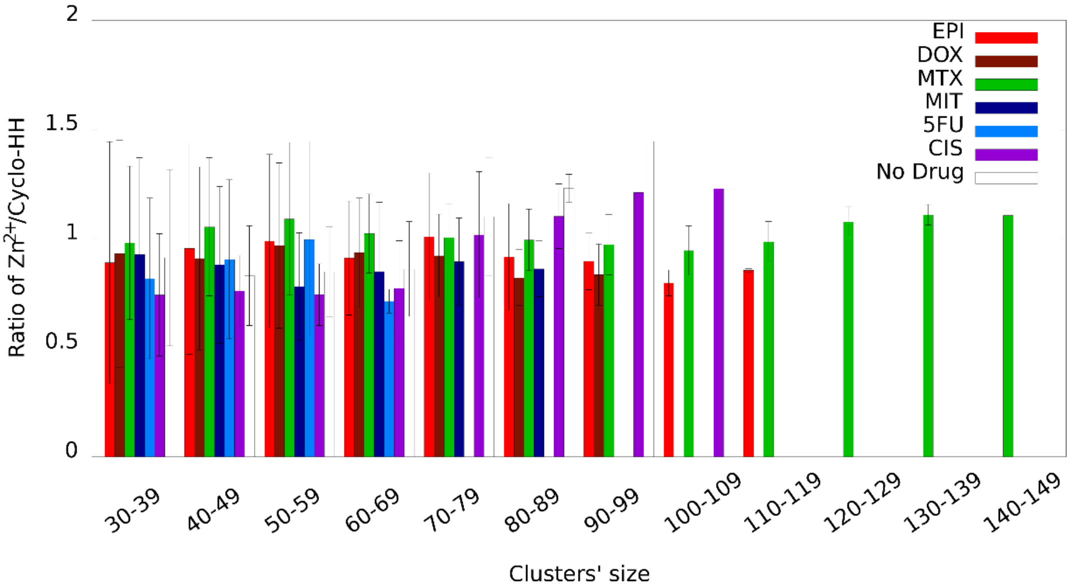

Figure S7. The ratio of Zn^{2+} divided by Cyclo-HH peptides in the clusters as a function of clusters’ size for clusters with: EPI (red), DOX (maroon), MTX (green), MIT (dark blue), 5FU (light blue), CIS (purple) and no drugs (colorless).

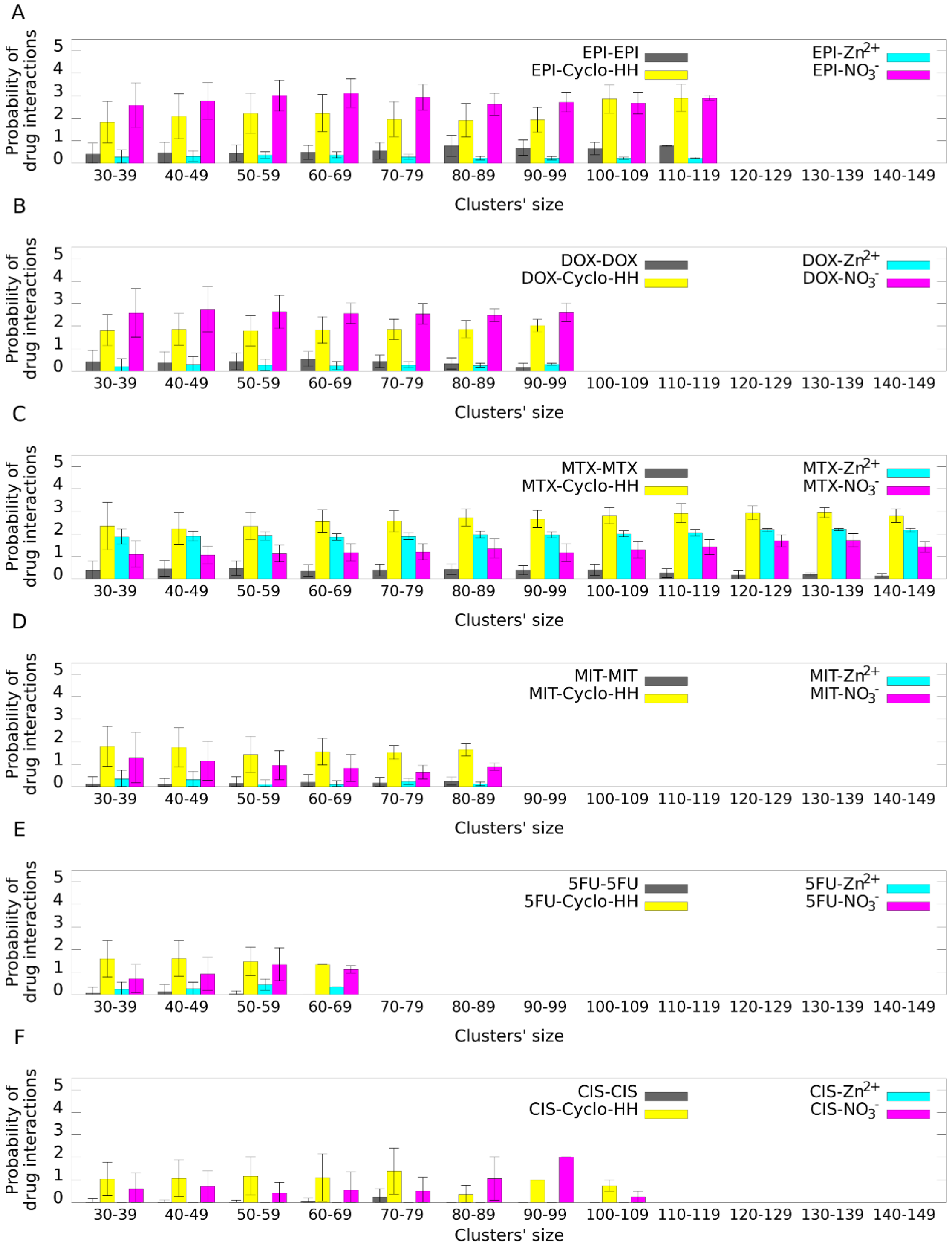

Figure S8. The probability of a drug to interact with: other drugs (gray), Cyclo-HH peptides (yellow), Zn^{2+} (cyan) and NO_3^- (violet), as a function of the clusters' size for clusters with: (A) EPI, (B) DOX, (C) MTX, (D) MIT, (E) 5FU and (F) CIS.

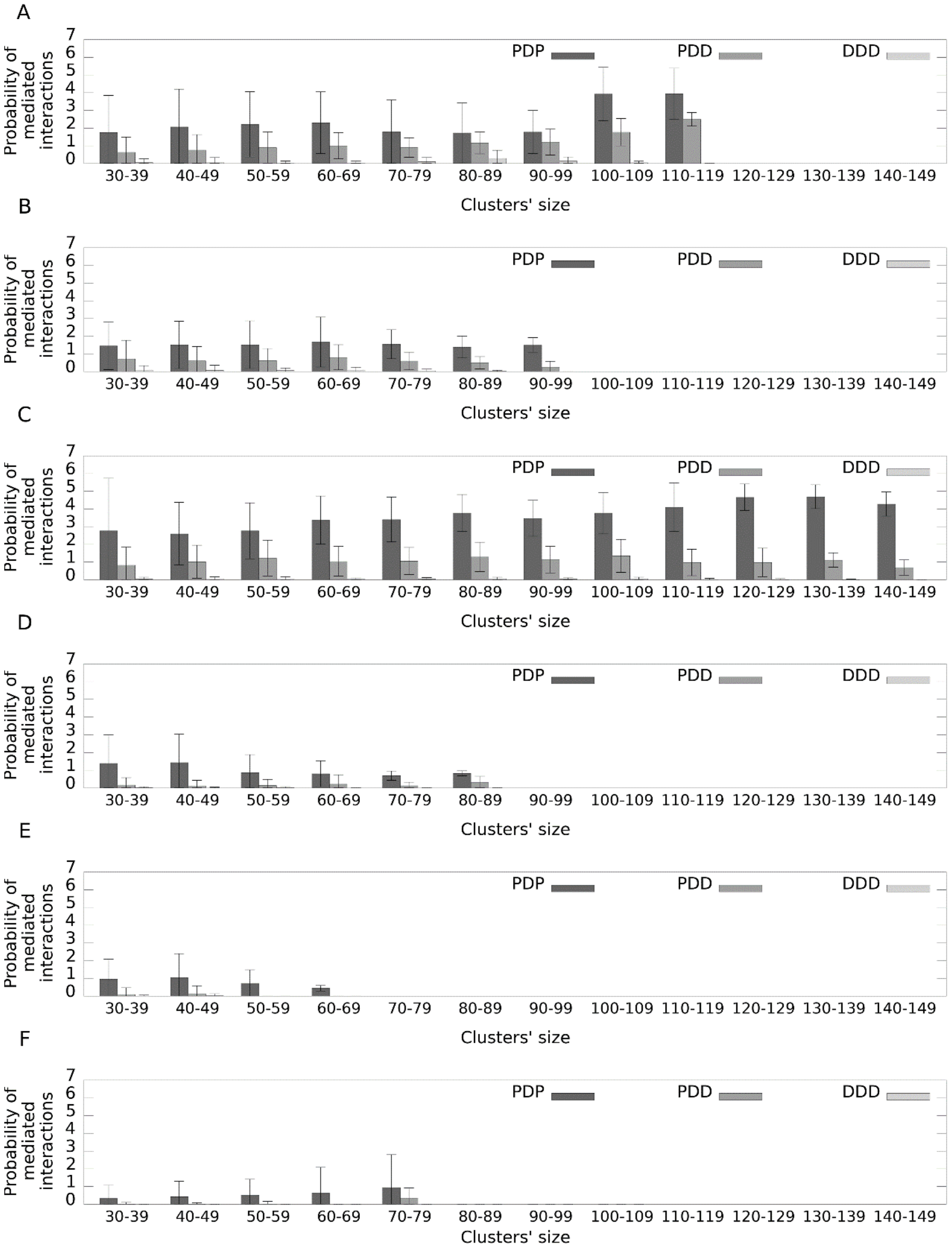

Figure S9. The probability of a drug(D) to mediate interactions with Cyclo-HH peptides (P) and/or other drugs (D) as a function of the clusters' size for clusters with: (A) EPI, (B) DOX, (C) MTX, (D) MIT, (E) 5FU and (F) CIS.

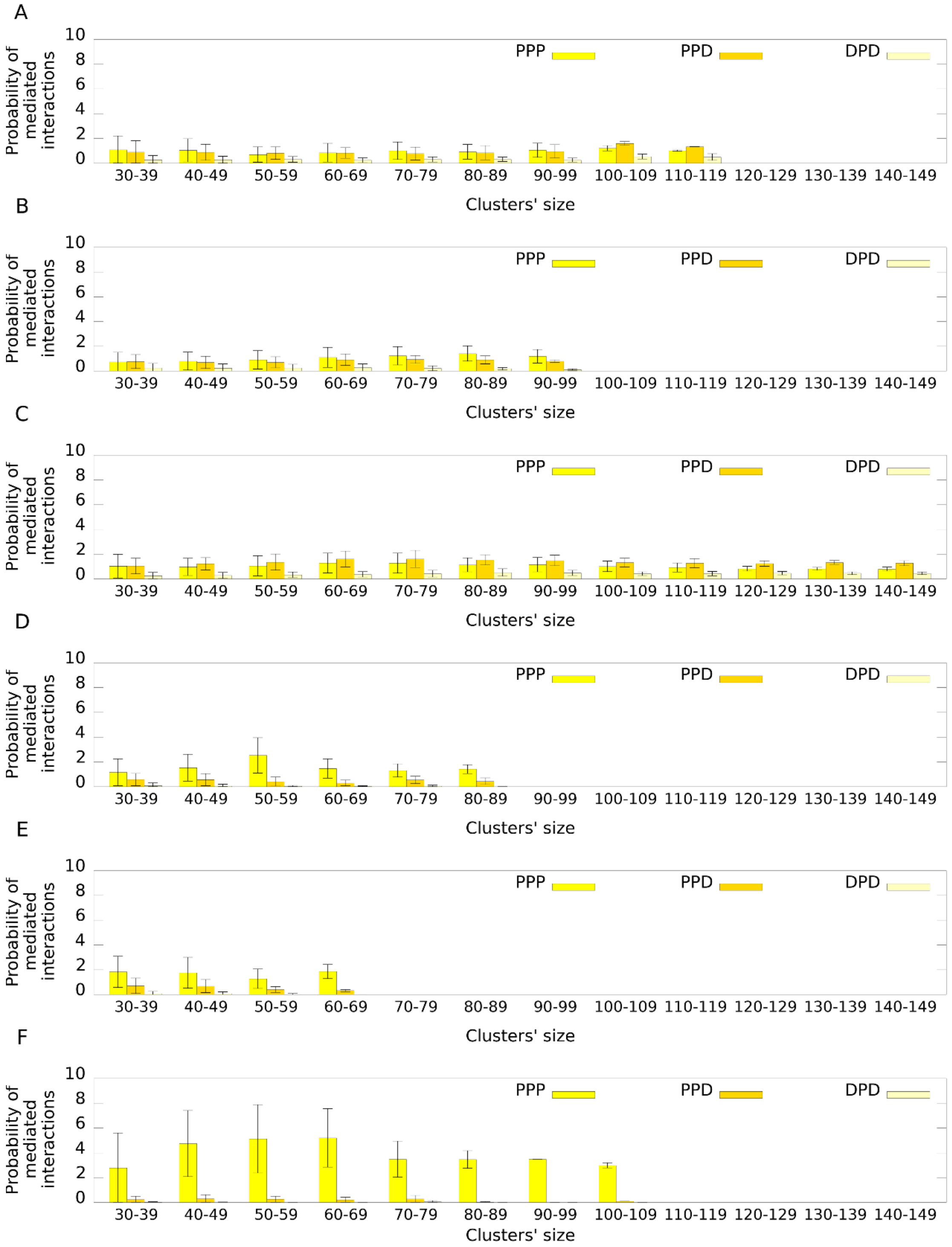

Figure S10. The probability of a Cyclo-HH peptide (P) to mediate interactions with other Cyclo-HH peptides (P) and/or drugs (D) as a function of the clusters' size for clusters with: (A) EPI, (B) DOX, (C) MTX, (D) MIT, (E) 5FU and (F) CIS

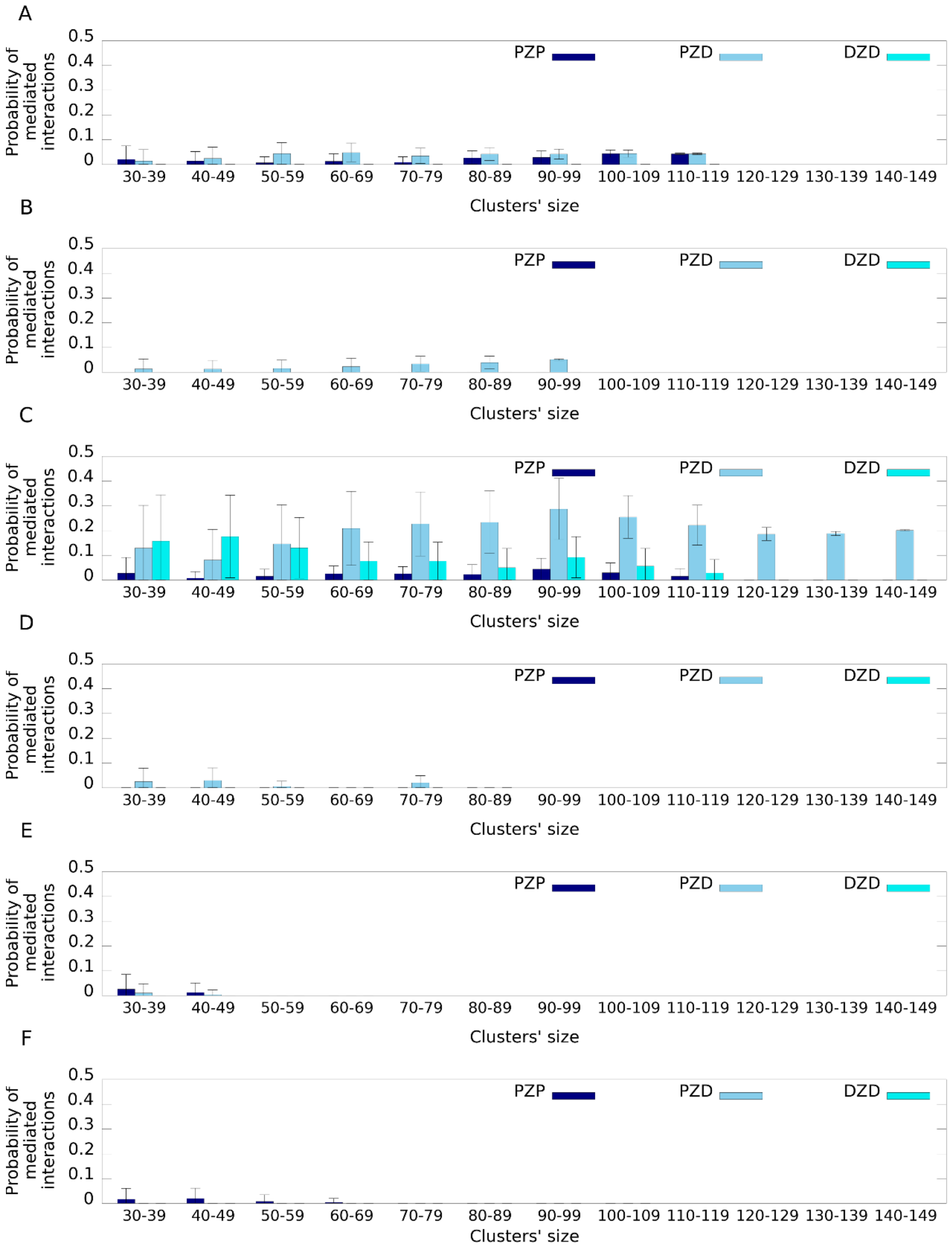

Figure S11. The probability of a Zn^{2+} (Z) to mediate interactions with Cyclo-HH peptides (P) and/or drugs (D) as a function of the clusters' size for clusters with: (A) EPI, (B) DOX, (C) MTX, (D) MIT, (E) 5FU and (F) CIS.

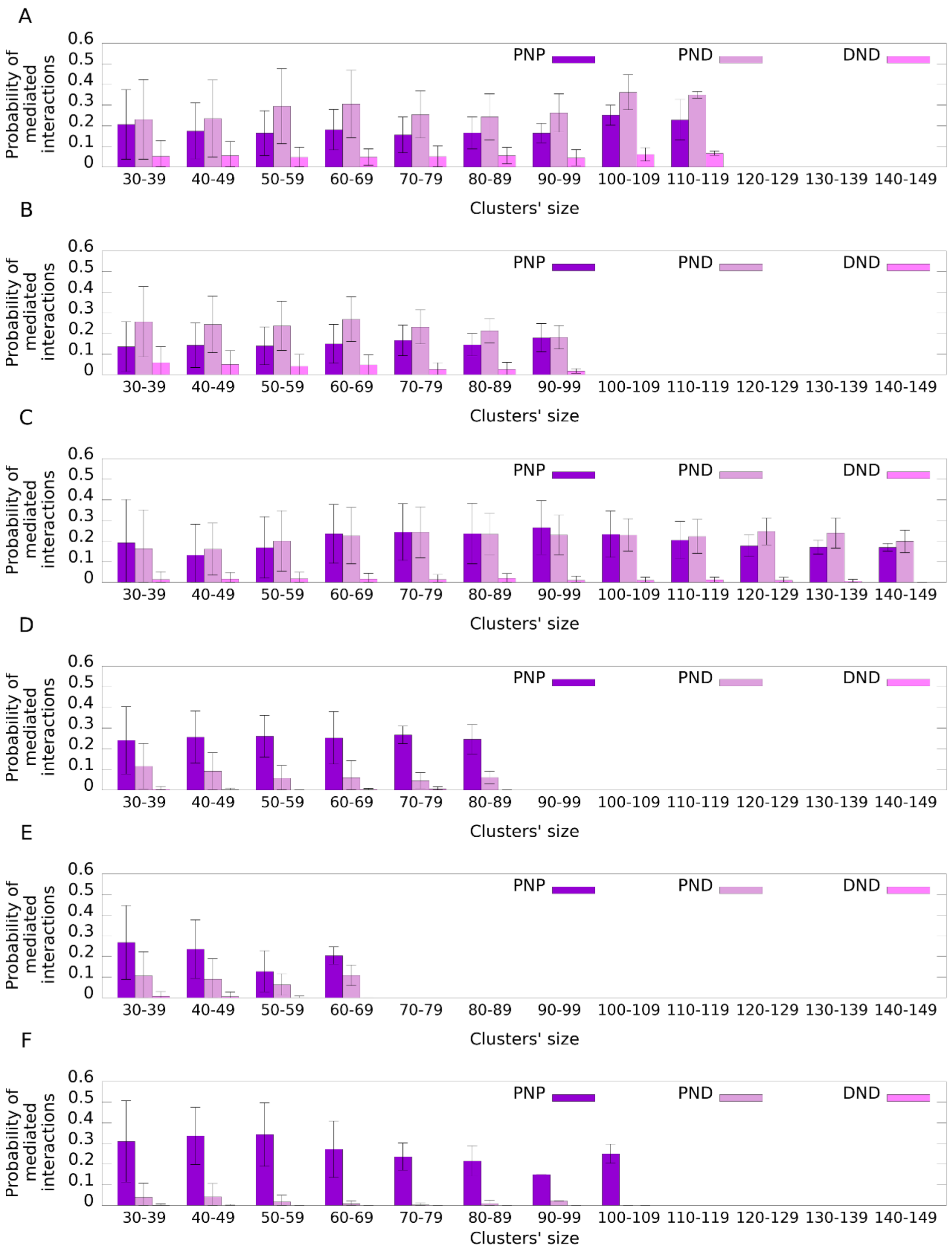

Figure S12. The probability of NO_3^- (N) to mediate interactions with Cyclo-HH peptides (P) and/or drugs (D) as a function of the clusters' size for clusters with: (A) EPI, (B) DOX, (C) MTX, (D) MIT, (E) 5FU and (F) CIS.

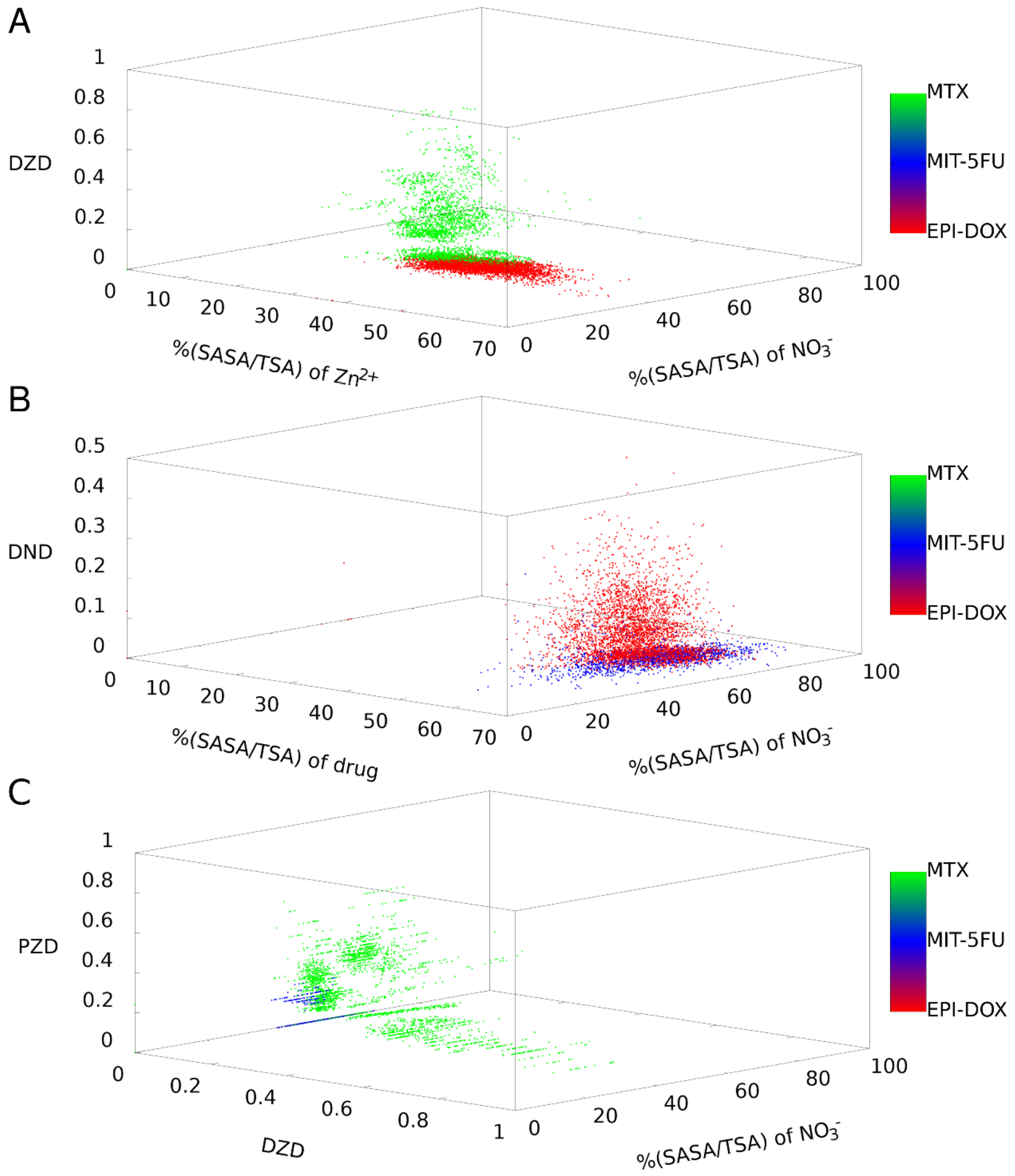

Figure S13. The three main features differ according to the SVM model, the three classes. (A) For the binary system of [EPI-DOX] class (red) vs [MIT-5FU] class (blue) [x-axis, y-axis, z-axis] = [%(SASA)/(TSA) of Zn^{2+}, %(SASA)/(TSA) of NO_3^-, DZD]. (B) For the binary system of [EPI-DOX] class (red) vs [MTX] class (green):) [x-axis, y-axis, z-axis] = [%(SASA)/(TSA) of Zn^{2+}, %(SASA)/(TSA) of drug, DND]. (C) For the binary system of [MIT-5FU] class (blue) vs [MTX] class (green):) [x-axis, y-axis, z-axis] = [DZD, %(SASA)/(TSA) of NO_3^-, PZD].

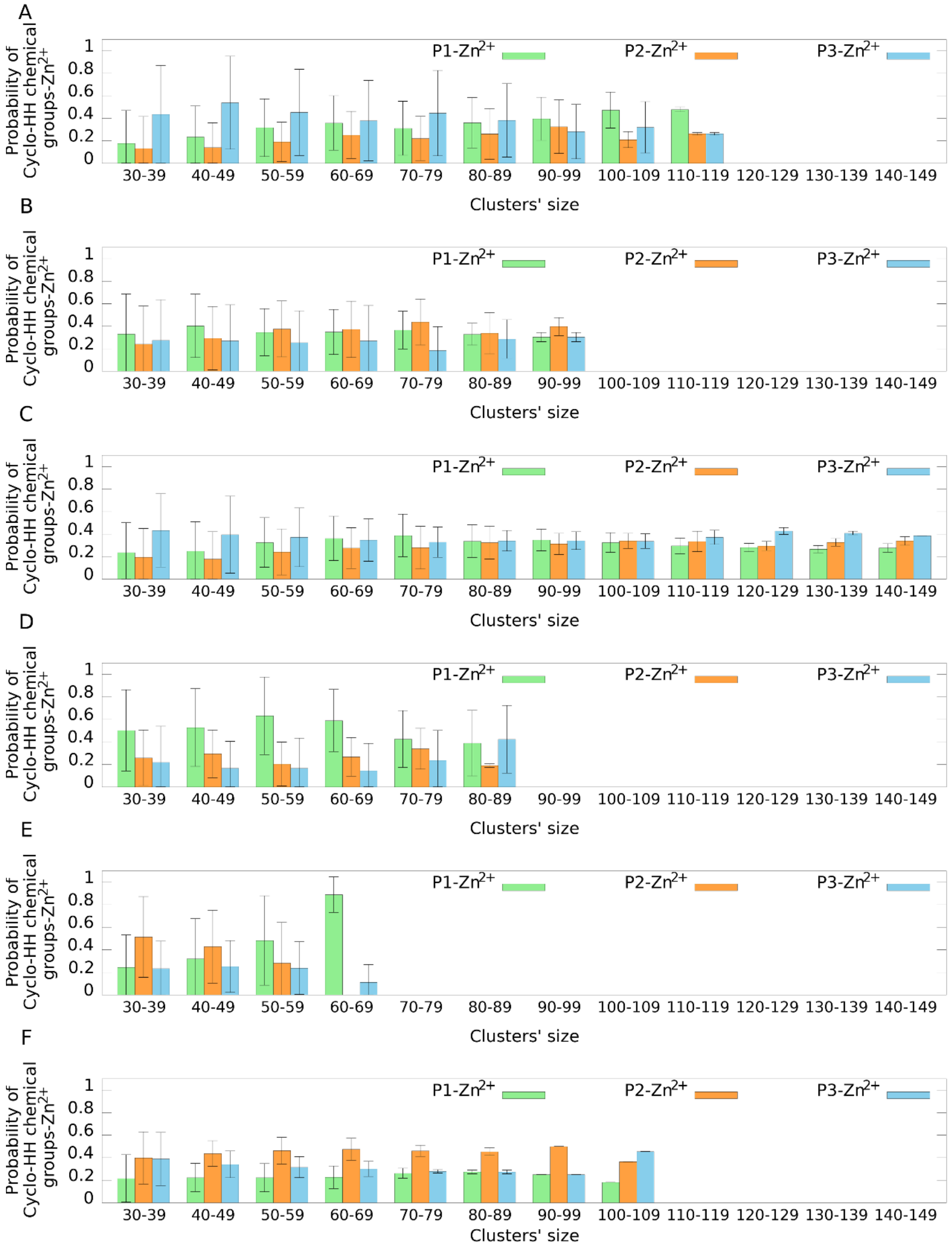

Figure S14. The probability of each Cyclo-HH chemical group (Figure S1) to interact with a Zn^{2+} as a function of clusters' size for clusters with: (A) EPI, (B) DOX, (C) MTX, (D) MIT, (E) 5FU and (F) CIS.

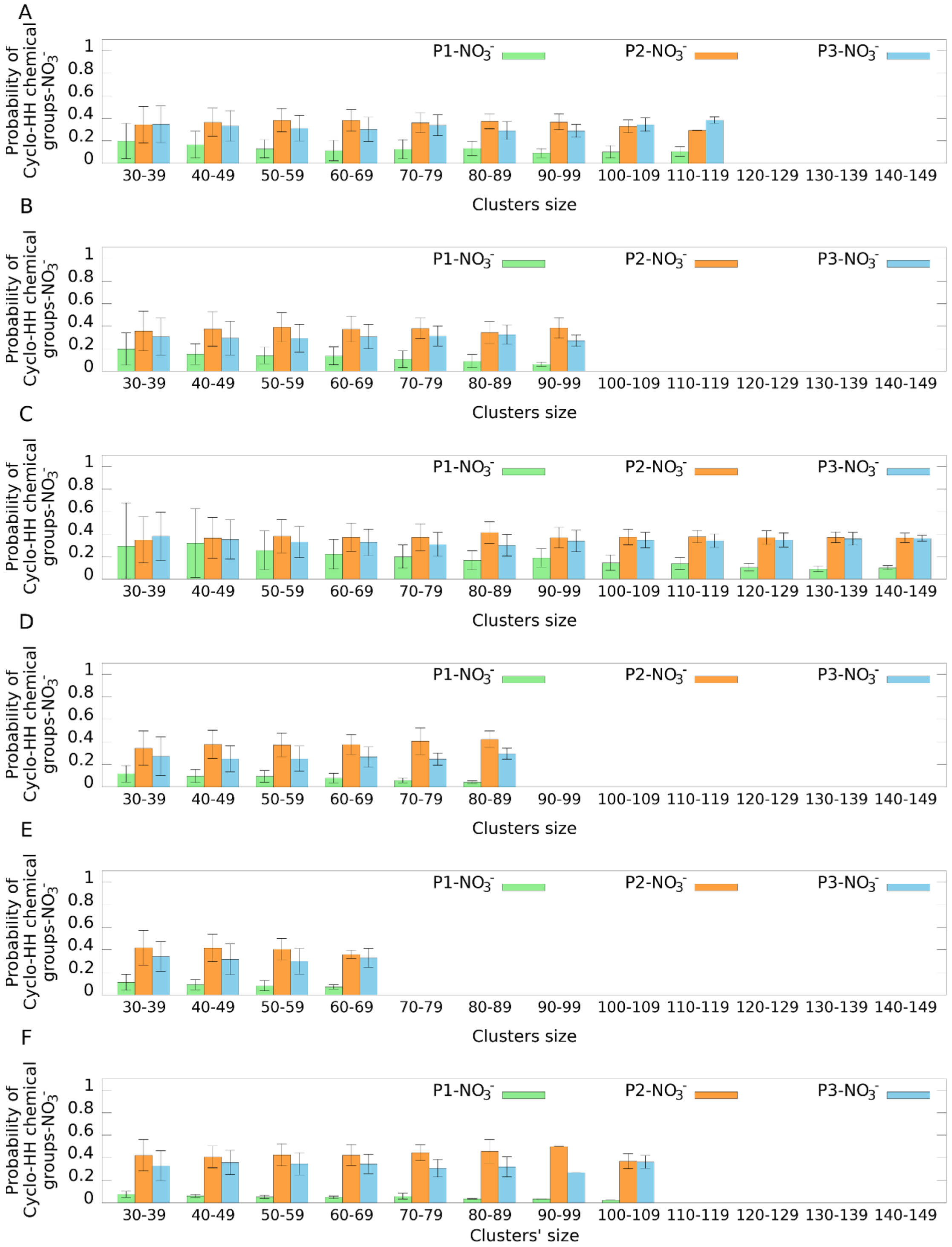

Figure S15. The probability of each Cyclo-HH chemical group (Figure S1) to interact with a NO_3^- as a function of clusters' size for clusters with: (A) EPI, (B) DOX, (C) MTX, (D) MIT, (E) 5FU and (F) CIS.

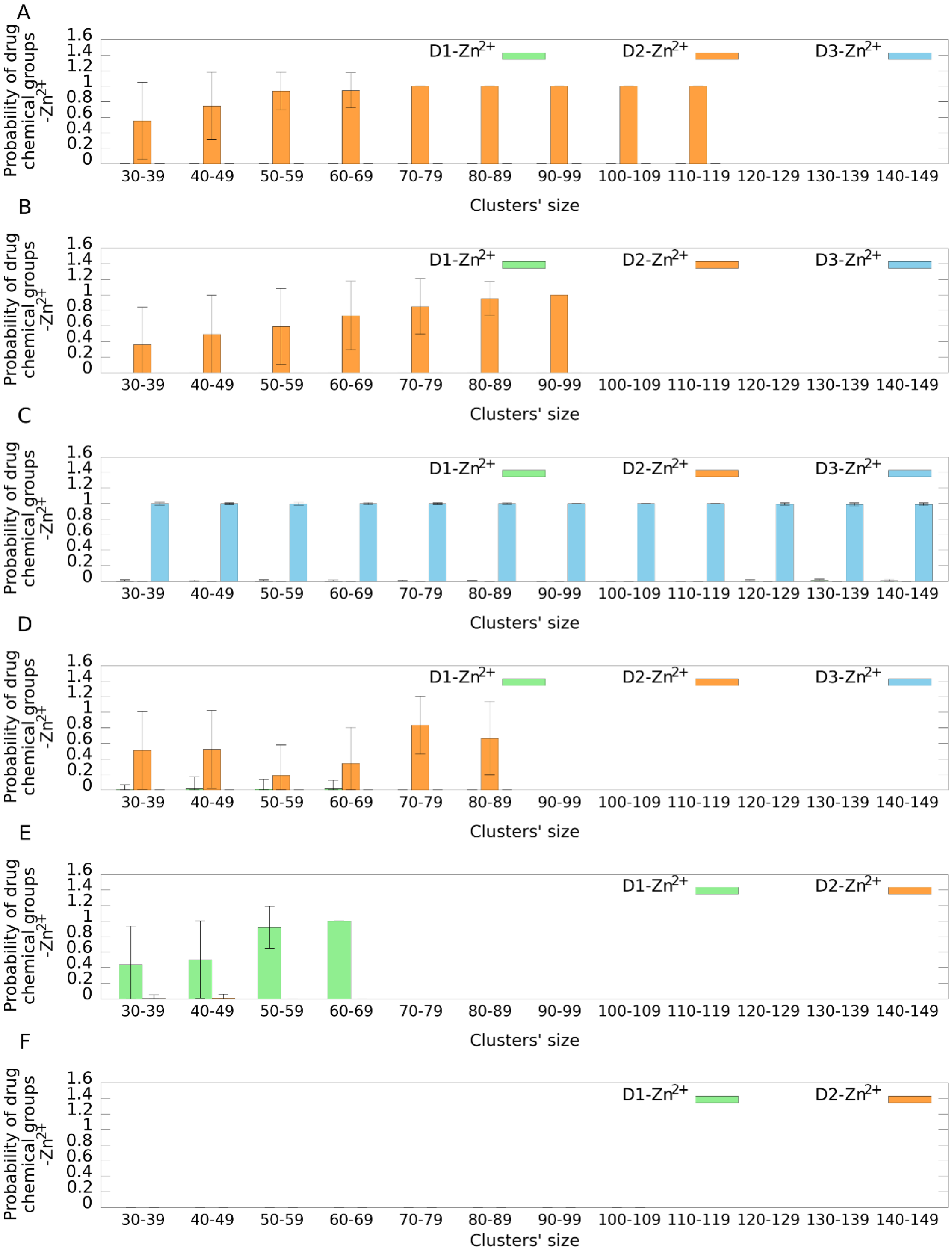

Figure S16. The probability of each drug chemical group (Figure S1) to interact with a Zn^{2+} as a function of clusters' size for clusters with: (A) EPI, (B) DOX, (C) MTX, (D) MIT, (E) 5FU and (F) CIS.

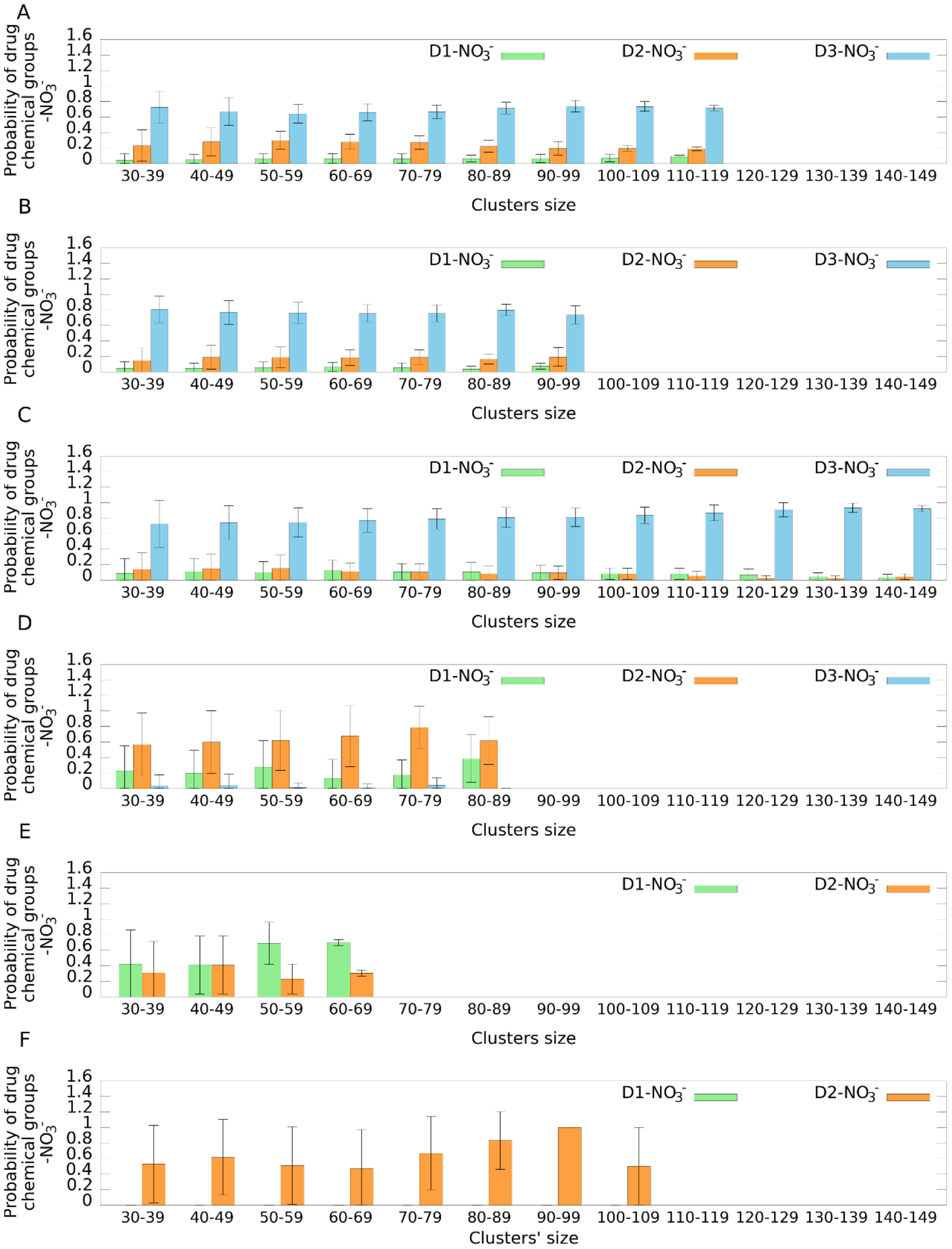

Figure S17. The probability of each drug chemical group (Figure S1) to interact with a NO_3^- as a function of clusters' size for clusters with: (A) EPI, (B) DOX, (C) MTX, (D) MIT, (E) 5FU and (F) CIS.

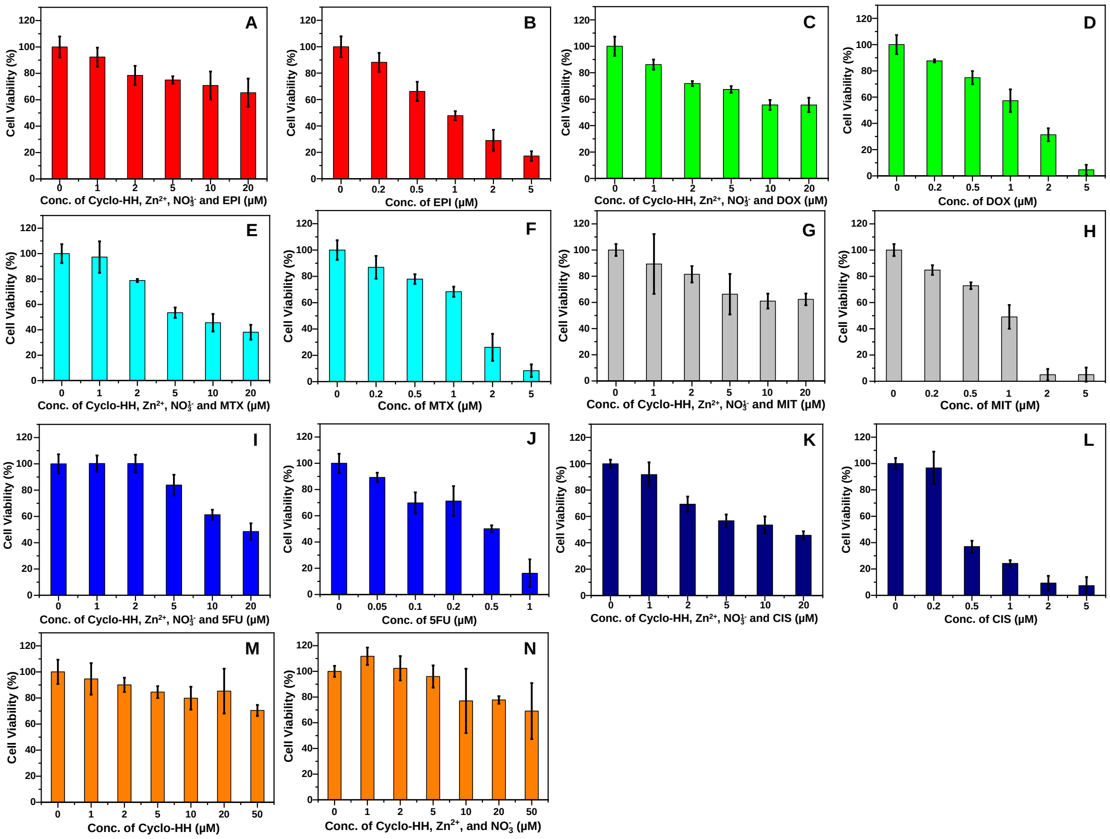

Figure S18. MTT assays examined the in vitro cell viability of (A) co-assembly of Cyclo-HH, Zn^{2+}, and NO_3^- with EPI, (B) EPI only (C) co-assembly of Cyclo-HH, Zn^{2+}, NO_3^- with DOX, (D) DOX, (E) co-assembly of Cyclo-HH, Zn^{2+}, NO_3^- with MTX, (F) pristine MTX, (G) co-assembly of Cyclo-HH, Zn^{2+}, and NO_3^- with MIT, (H) pristine MIT, (I) co-assembly of Cyclo-HH, Zn^{2+}, and NO_3^- with 5FU, (J) Pristine 5FU, (K) co-assembly of Cyclo-HH, Zn^{2+}, and NO_3^- with CIS, (L) pristine CIS drugs alone, (M) pristine Cyclo-HH, and (N) co-assembly of Cyclo-HH, Zn^{2+}, and NO_3^- nanostructure on on the Hela cell line.

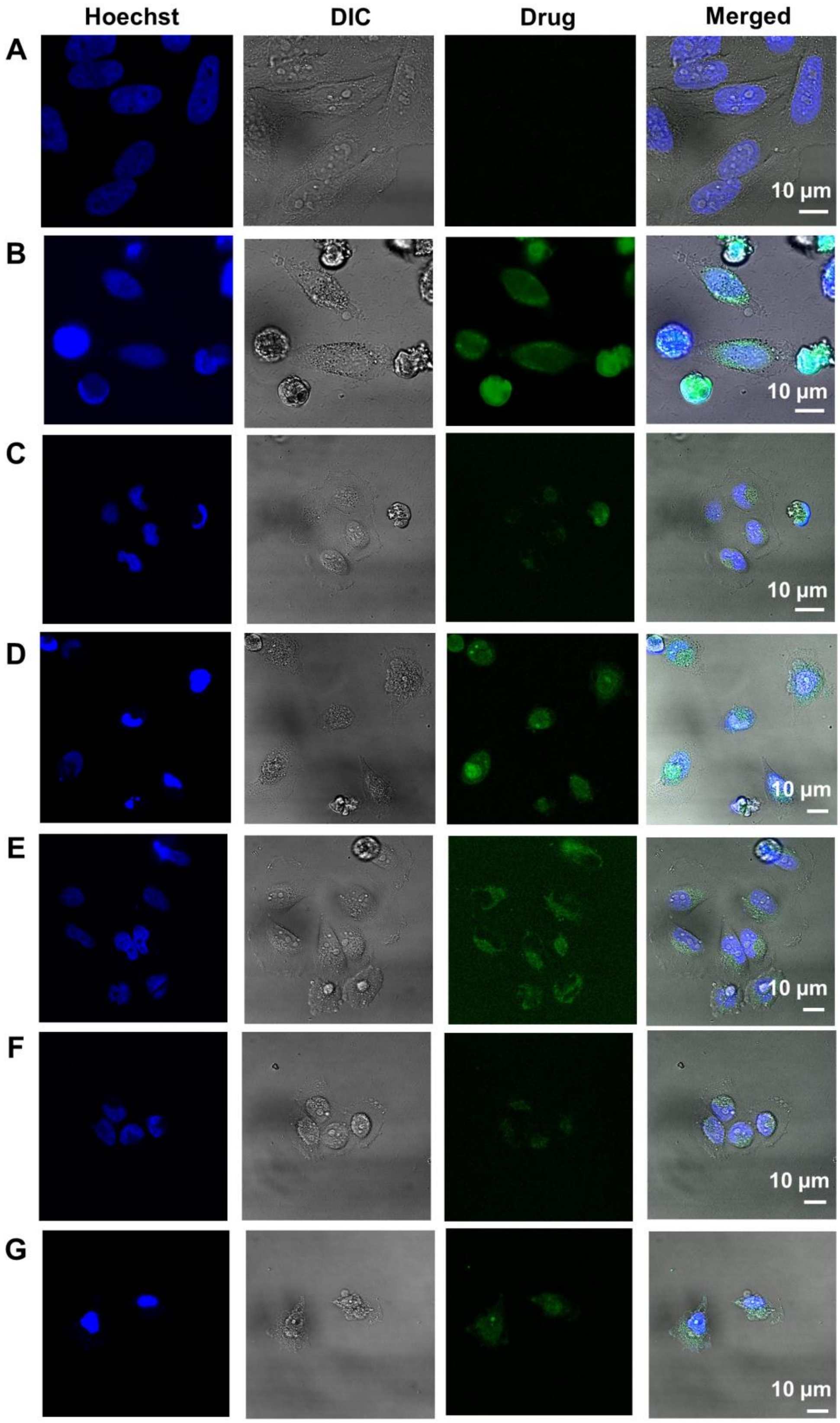

Figure S19. Live imaging of HeLa cells by confocal microscopy. (A) Control without any treatment. (B-G) after a 24-hour incubation with pristine drug only (B) EPI, (C) DOX, (D) MTX, (E) MIT, (F) 5FU, and (G) CIS.

Supporting References

1 Seeber M, Cecchini M, Rao F, Settanni G, Caflisch A. Wordom: a program for efficient analysis of molecular dynamics simulations. Bioinformatics. 2007;23(19):2625-7.

2 Seeber M, Felline A, Raimondi F, Muff S, Friedman R, Rao F, Caflisch A, Fanelli F. Wordom: a user-friendly program for the analysis of molecular structures, trajectories, and free energy surfaces. J Comput Chem. 2011;32(6):1183-94.

3 Brooks BR, Brooks CL 3rd, Mackerell AD Jr, Nilsson L, Petrella RJ, Roux B, Won Y, Archontis G, Bartels C, Boresch S, Caflisch A, Caves L, Cui Q, Dinner AR, Feig M, Fischer S, Gao J, Hodoscek M, Im W, Kuczera K, Lazaridis T, Ma J, Ovchinnikov V, Paci E, Pastor RW, Post CB, Pu JZ, Schaefer M, Tidor B, Venable RM, Woodcock HL, Wu X, Yang W, York DM, Karplus M. CHARMM: the biomolecular simulation program. J Comput Chem. 2009;30(10):1545-614.

4 Santos-Martins D, Forli S, Ramos MJ, Olson AJ. AutoDock4(Zn): an improved AutoDock force field for small-molecule docking to zinc metalloproteins. J Chem Inf Model. 2014;54(8):2371-9.

5 Buša J, Džurina J, Hayryan E, Hayryan S, Hu CK, Plavka J, Pokorný I, Skřivánek J, Wu MC. ARVO: A Fortran package for computing the solvent accessible surface area and the excluded volume of overlapping spheres via analytic equations. Computer Physics Communications. 2005;165(1):59–96.

6 Prakash P, Hancock JF, Gorfe AA. Binding hotspots on K-ras: consensus ligand binding sites and other reactive regions from probe-based molecular dynamics analysis. Proteins. 2015;83(5):898-909.

7 Bakan A, Nevins N, Lakdawala AS, Bahar I. Druggability Assessment of Allosteric Proteins by Dynamics Simulations in the Presence of Probe Molecules. J Chem Theory Comput. 2012;8(7):2435-2447.
